# Long noncoding RNA#61 synergizes with viral PA-X to augment pyroptosis and attenuate the virulence of highly pathogenic H5N1 influenza virus in mice

**DOI:** 10.1128/jvi.02214-25

**Published:** 2026-05-12

**Authors:** Xia Chen, Xinxin Zheng, Lei Zhang, Xinxin Cai, Xinping Hong, Siyu Hou, Xuran Ma, Zenglei Hu, Min Gu, Xiaoquan Wang, Ruyi Gao, Shunlin Hu, Yu Chen, Xiaowen Liu, Daxin Peng, Xiufan Liu, Jiao Hu

**Affiliations:** 1 Key Laboratory of Avian Bioproducts Development, College of Veterinary Medicine, Yangzhou University 614704 , Yangzhou, People's Republic of China; 2 Jiangsu Co-Innovation Center for Prevention and Control of Important Animal Infectious Diseases and Zoonosis, College of Veterinary Medicine, Yangzhou University 614704 , Yangzhou, People's Republic of China; 3 Key Laboratory of Prevention and Control of Biological Hazard Factors (Animal Origin) for Agri-food Safety and Quality, Ministry of Agriculture of China, College of Veterinary Medicine, Yangzhou University 614704 , Yangzhou, People's Republic of China; 4 Joint International Research Laboratory of Agriculture and Agri-Product Safety, Ministry of Education of China, Yangzhou University 38043, Yangzhou, People's Republic of China; University of Freiburg, Freiburg, Germany

**Keywords:** highly pathogenic H5N1 influenza virus, long non-coding RNA, PA-X, pyroptosis, antiviral effect

## Abstract

Long non-coding RNAs (LncRNAs) play pivotal regulatory roles in various biological processes, notably in immune regulation and viral infection. We previously identified the broad anti-influenza activity for LncRNA#61. Here, we further investigate the mechanism underlying its antiviral effect, both *in vitro* and *in vivo*. Using a lipid-nanoparticle-based delivery strategy, LncRNA#61 was successfully delivered into mice and effectively attenuated the replication and virulence of the highly pathogenic H5N1 influenza virus. Integrative transcriptomic analysis revealed that forced expression of LncRNA#61 markedly activated lipid metabolism, cell death, and Ragulator-Rag-mTORC1 pathways. Quantitative reverse transcription PCR analysis and a targeting metabolic assay further confirmed that LncRNA#61 is actively involved in regulating these pathways. Subsequent functional studies demonstrated that LncRNA#61 consistently enhances GSDMD-mediated pyroptosis both in murine LET-1 and canine MDCK cells. Notably, such pyroptosis was found to restrict H5N1 influenza virus replication. Intriguingly, ectopic expression of viral PA-X protein enhanced antiviral activity of LncRNA#61 both *in vitro* and *in vivo*. Mechanistically, PA-X interacts with LncRNA#61 and promotes LncRNA#61-mediated pyroptosis via a RagA-dependent reactive oxygen species pathway. Collectively, we here propose a novel model in which viral and host factors cooperate to activate a pro-death antiviral pathway. Our findings not only advance the fundamental knowledge of virus–host interactions but also cross-link cell death, innate immunity, and metabolic regulation, pinpointing novel therapeutic targets against influenza.

**IMPORTANCE:**

A current priority in anti-influenza research is developing broad-spectrum, host-directed therapeutics with low resistance risk. Here, we reveal that LncRNA#61-induced pyroptosis exerts an antiviral effect by restricting H5N1 virus replication both *in vitro* and *in vivo*, highlighting a novel cooperative virus–host interaction that enhances antiviral immunity. Key contributions include the following: (i) identifying pyroptosis as a direct executioner mechanism that restricts H5N1 virus infection; (ii) revealing the unexpected role of forced expression of viral PA-X in augmenting antiviral activity of host LncRNA#61; and (iii) deciphering that LncRNA#61 interacts with PA-X and synergistically promotes GSDMD-mediated pyroptosis through a RagA‑ROS signaling cascade. Collectively, our work elucidates a novel antiviral mechanism wherein host LncRNA and viral protein co-opt the RagA-ROS-GSDMD axis to drive pyroptosis and inhibit viral replication. This discovery innovatively establishes a novel connection among viral pathogenesis, host cell death, and cellular metabolism, offering a fresh, integrative perspective for future studies on host-directed antiviral strategies.

## INTRODUCTION

Influenza A virus (IAV) remains a critical global public health threat. Notably, avian influenza viruses (AIVs), particularly H5N1/N6/N8, H6N1, H7N2/N3/N7/N9, H9N2, and H10N7/N8 subtypes, not only threaten the sustainable development of the poultry industry but also exhibit potential zoonotic threat. These viruses cause severe human infection, including acute respiratory distress syndrome (ARDS), multiple organ failure (MOF), and even mortality. Since 2020, H5N1 AIVs have exhibited rapid intercontinental spread, with massive poultry culling events reported, and unprecedented terrestrial and marine mammalian spillover events. Currently, over 50 mammalian species, including dairy cows, farmed mink, sea lions, foxes, and cats, can be infected by the H5N1 virus (1, 2). H5N1 virus infection also brought out ecological crises through decreasing wild bird populations (e.g., South American pelicans, European gulls), posing endangerment pressure on endangered species (e.g., Antarctic penguins), and disrupting the marine trophic cascade (e.g., the sea lion massive mortality events). Alarmingly, the H5N1 virus’s progressive mammalian adaptation raises concerns about pandemic potential through efficient human-to-human transmission (3). Collectively, H5N1 and other AIVs continually pose significant threats to the development of animal husbandry, ecological stability, and human health.

Current influenza control strategies primarily rely on vaccines and antiviral drugs. However, the effectiveness of vaccines is often compromised by the antigenic drift, which is mainly driven by the error-prone nature of the viral RNA polymerase. Consequently, the antigenic evolution of the virus necessitates the continuous updating of vaccines. On the other hand, existing antivirals mainly target viral proteins (direct-acting antiviral [DAA] drugs), such as inhibitors of neuraminidase (NA), the M2 ion channel, or polymerase complex subunits. Such DAA drugs have limitations of drug resistance and low broad-antiviral activity. In contrast, the host-directed antiviral (HTA) strategy circumvents such defects by targeting the conserved cellular pathways that are hijacked by the viral replication machinery (4–6). Therefore, broad-spectrum anti-influenza drugs with a low risk of inducing resistance are urgently needed. However, novel HTA targets and their underlying antiviral mechanisms remain underexplored.

Traditional screening of antiviral targets prioritizes the protein-coding genes, yet over 98% of the mammalian genomes are transcribed into noncoding RNAs (ncRNAs), predominantly the long noncoding RNAs (LncRNAs). Mounting evidence establishes LncRNAs as the master regulators of diverse biological processes, including cell proliferation, differentiation, metastasis, signal transduction, cell cycle control, and immune and metabolic homeostasis (7, 8). These molecules orchestrate gene expression through epigenetic remodeling, transcriptional modulation, and post-transcriptional regulation (9–11). Meanwhile, LncRNAs play increasingly recognized roles in viral pathogenesis, underscoring their potential as antiviral targets, as evidenced by various research on theiler’s murine encephalomyelitis virus (TMEV) (11), severe acute respiratory syndrome coronavirus-2 (SARS-CoV-2) (12), hantavirus (HTNV) (13), hepatitis C virus (HCV) (14), human immunodeficiency virus (HIV) (15), hepatitis B virus (HBV) (16), Japanese encephalitis virus (JEV) (17), and vesicular stomatitis virus (VSV) (18). During IAV infection, LncRNAs modulate antiviral immunity through multifaceted mechanisms. For example, acting as molecular scaffolds (19, 20) and signals (19, 21), trafficking guides (22, 23), or decoy RNAs (24, 25). In addition, LncRNAs are actively involved in regulating immune pathways via chromatin remodeling (26, 27), transcriptional control (28), or post-transcriptional modifications (29). Conversely, IAV can also hijack specific host LncRNAs to facilitate its own transcription and translation (29). Despite these individual findings, the broader regulatory networks of LncRNAs in influenza pathogenesis and their consequent therapeutic potential are still largely uncharted.

Pyroptosis, a proinflammatory form of programmed cell death, has garnered increasing interest for its roles in viral infection, cancer, and autoimmune diseases. Current evidence has revealed its context-dependent functions in viral pathogenesis, driving both beneficial effects and pathological inflammation. For instance, herpes simplex virus (HSV) suppresses gasdermin E (GSDME)-mediated pyroptosis to enhance replication (30), whereas coxsackievirus B3 (CVB3) induces pyroptosis to facilitate early viral dissemination (31). In the context of influenza, caspase-6 promotes H1N1 virus-induced pyroptosis, apoptosis, and necroptosis, contributing to host protection in mice (32). In contrast, pyroptosis exacerbates the pulmonary cytokine storm and lethality in H7N9-infected mice (33) and constitutes the primary mechanism of alveolar cell death in lethal H5N1 infection in macaques (34). Interestingly, pyroptotic cells release host factors with contrasting effects: not only pro-inflammatory mediators such as IL-1β but also pro-repair molecules like oxylipins and metabolites (35, 36). Despite these important findings, direct evidence defining how pyroptosis specifically influences influenza virus replication and pathogenesis both *in vitro* and *in vivo* still remains inadequately defined.

Our previous deep sequencing analysis of mouse lungs infected with H5N1 AIVs identified a functionally promising lncRNA, LncRNA#61 (37). Functional studies demonstrated that LncRNA#61 significantly inhibits replication of multiple IAV subtypes (38), including human H1N1 as well as avian H3N2/N8, H4N6, H5N1, H6N2/N8, H7N9, H8N4, H10N3, and H11N2/N6/N9 viruses. Mechanistically, the broad antiviral activity is attributed primarily to its four long stem-loop structures, which contribute to inhibiting viral polymerase activity and the nuclear aggregation of key polymerase components. However, the precise mechanism driving antiviral activity of LncRNA#61 was not fully understood. Herein, we report that LncRNA#61 executes its antiviral function by directly enhancing pyroptosis in both cellular and animal models. Intriguingly, this host defense pathway is amplified, rather than subverted, by the viral PA-X protein. We further elucidate a novel cooperative mechanism wherein PA-X interacts with LncRNA#61 to drive pyroptosis specifically via the RagA-ROS-GSDMD axis. Collectively, this study not only defines a novel lncRNA-mediated antiviral paradigm but also reveals an unexpected role for a viral factor in augmenting host defense, thereby providing fresh mechanistic insights and potential therapeutic targets for combating influenza.

## RESULTS

### LncRNA#61 limits H5N1 influenza virus infection in mice

**Fig 1:**
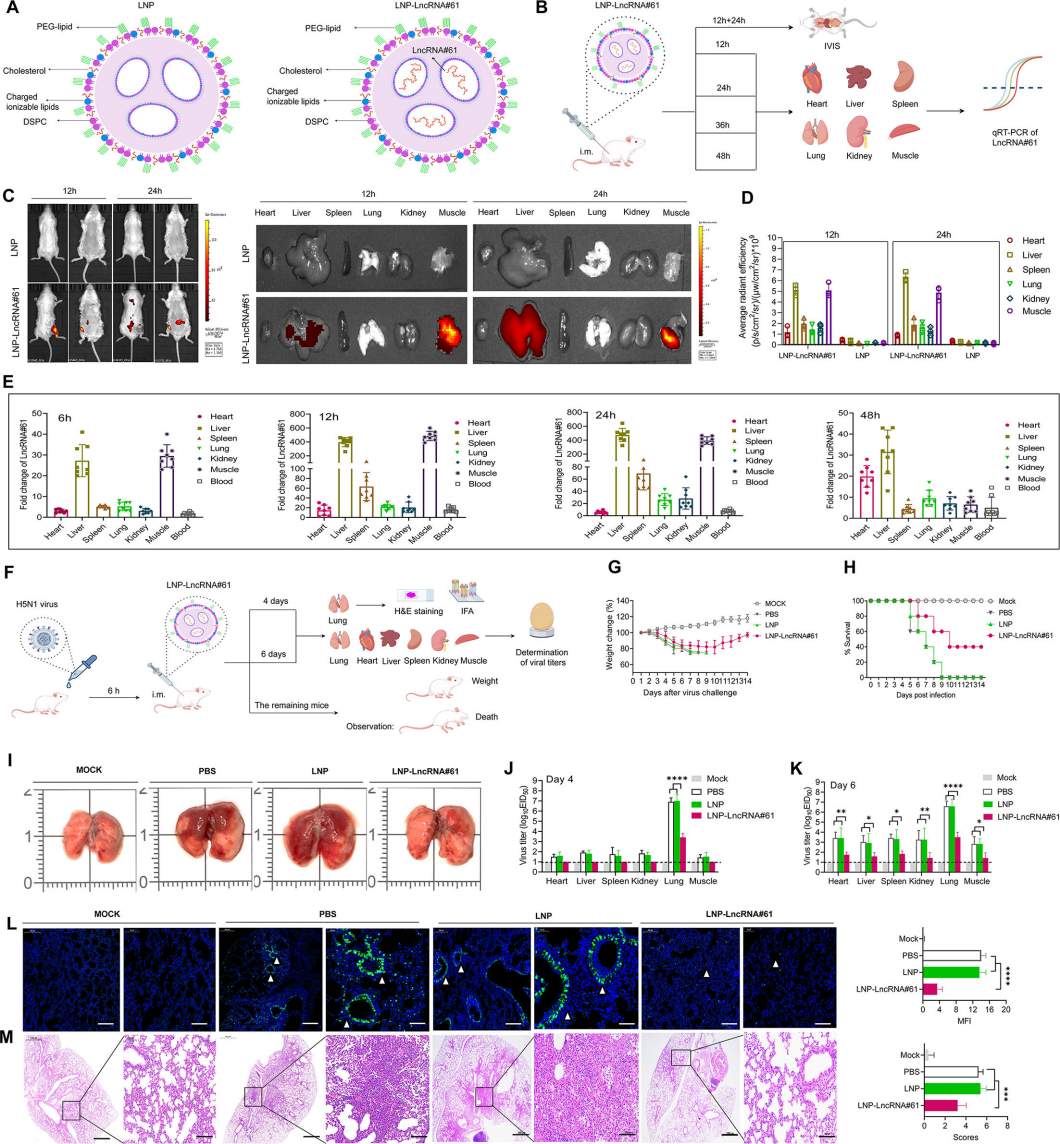
LncRNA#61 limits H5N1 infection in mice. (**A**) Schematic of the LNP-LncRNA#61 construct. LNP-LncRNA#61 was prepared using a microfluidic mixing method. Briefly, the lipid mixtures were dissolved in anhydrous ethanol using charged ionizable lipids, 1,2-distearoyl-sn-glycero-3-phosphocholine (DSPC), cholesterol, and PEG-lipids to form the organic phase. (**B**) Schematic diagram of the investigation of the *in vivo* distribution of LNP-LncRNA#61 in mice. (**C**) *In vivo* distribution of LNP-LncRNA#61 in mice and mouse organs. *In vivo* imaging was performed at 12 h and 24 h post-inoculation. Following imaging, major organs were collected at the corresponding time points for *ex vivo* fluorescence measurement of LncRNA#61 bio-distribution. (**D**) The fluorescence intensity of the mouse organs in panel C. Data are shown as the means ± standard deviation (SD) (*n* = 3). (**E**) Mouse organs were collected at the indicated time points for qRT-PCR analysis of LncRNA#61. Data are shown as the means ± SD (*n* = 8). (**F**) Schematic of the *in vivo* antiviral efficacy evaluation of LNP-LncRNA#61. (**G**) Body weight and (**H**) survival rate of the mice were monitored daily for 14 days (*n* = 5). (**I**) Necropsy was performed for observation of gross lung pathology at 6 days post-infection (p.i.). (**J–K**) Viral replication in mouse organs at day 4 p.i. (**J**) and at day 6 p.i. (**K**). Data are means ± SD (*n* = 3). Statistical significance was calculated by one-way ANOVA with Tukey’s multiple comparison test, *P < 0.05, **P < 0.01, ****P < 0.0001. (**L**) Immunostaining of the lung tissues with an influenza virus NP-specific monoclonal antibody and quantification of the mean fluorescence intensity (MFI) at 6 days p.i. Scale bar: 200 µm (left) and 100 µm (right). The white triangle indicates the positive area for the NP protein. Data are shown as means ± SD and analyzed by one-way analysis of variance (ANOVA) with Tukey’s multiple comparison test, ****P < 0.0001. (**M**) Hematoxylin and eosin (H&E) staining and pathological scoring of the lung tissues from different groups of mice at 6 days p.i. Scale bar: 1,000 µm (left) and 100 µm (right). Data are shown as means ± SD and analyzed by one-way ANOVA with Tukey’s multiple comparison test, ***P < 0.001.

Nonviral delivery of noncoding RNA *in vivo*, including the lipid nanoparticle (LNP)-encapsulated delivery strategy, shows good promise for encapsulation efficacy and stability *in vivo* (39–41). To determine the antiviral effect of the long noncoding RNA LncRNA#61 *in vivo*, we developed a LNP-loaded strategy for delivery of LncRNA#61 (named as LNP-LncRNA#61) in mice (Fig. 1A). To evaluate the *in vivo* delivery efficiency of LNP, we covalently labeled LncRNA#61 with Cy5.5 dye. The labeled LncRNA#61 was then encapsulated in LNP and administered to mice via multiple intramuscular injections in the hind leg (Fig. 1B). The *in vivo* imaging showed that LNP-LncRNA#61 was primarily localized in the liver and muscle at 12 and 24 h post-delivery (p.d.) (Fig. 1C). However, more sensitive quantitative analysis using Living Image software revealed its detectable distribution not only in these organs but also in the heart, spleen, lungs, and kidneys at both time points (Fig. 1D). Quantitative reverse transcription-PCR (qRT-PCR) analysis results further confirmed accumulation of LncRNA#61 in these organs at 6, 12, 24, and 48 h p.d. (Fig. 1E). Notably, the expression of LncRNA#61 peaked at 24 h p.d., with the highest level detected in the liver, followed by muscle, spleen, lung, kidneys, blood, and heart.

We then examined the antiviral effect of LncRNA#61 in mice. PBS, LNP, or LNP-LncRNA#61 was delivered into mice at 6 h post-infection (p.i.) with the A/Chicken/Jiangsu/k0402/2010 H5N1 (CK10) influenza A virus (Fig. 1F). Mice were measured daily for their body weights and survival. No weight loss or mortality was observed in the mock control mice throughout the 14-day observation period (Fig. 1G and H). In contrast, mice treated with LNP or PBS followed by virus infection exhibited significant weight loss, and all died within 9 days. By comparison, 40% of the mice that received LNP-LncRNA#61 prior to viral challenge survived until the end of the experiment (Fig. 1H). Gross pathological assessment indicated that lungs from LNP or PBS-treated mice were characterized by severe red blood cell extravasation, whereas this pathology was markedly alleviated in mice inoculated with LNP-LncRNA#61 (Fig. 1I). Virological analysis showed that on 4 days p.i., H5N1 virus can be recovered from heart, liver, spleen, kidneys, and muscle of the LNP or PBS-treated mice but not from the corresponding organs of the LNP-LncRNA#61-treated mice (Fig. 1J). Moreover, at this time point, lung viral titers were significantly lower in the LNP-LncRNA#61 group than those of LNP or PBS-inoculated mice. Similarly, compared with these two groups, significantly reduced viral titers were observed in all the tested organs of the LNP-LncRNA#61-inoculated group at 6 days p.i. (Fig. 1K). In addition, there was no significant difference in viral titers between LNP-inoculated and PBS-treated mice, confirming that the blank LNP formulation itself does not significantly perturb viral replication. These findings were correlated well with the higher levels of viral NP protein expression in the lungs of the LNP or PBS-treated mice compared to the LNP-LncRNA#61-treated group (Fig. 1L). Supporting these data, histological analysis demonstrated attenuated alveolar damage and interstitial inflammatory cell infiltration in the lungs of LNP-LncRNA#61-treated mice at this time point, which corresponded to significantly lower histopathological scores (Fig. 1M). Taken together, these findings suggest that LncRNA#61 effectively attenuates H5N1 virus replication and the associated pathogenesis *in vivo*.

### LncRNA#61 enhances lipid metabolism and cell death-related response during virus infection

**Fig 2:**
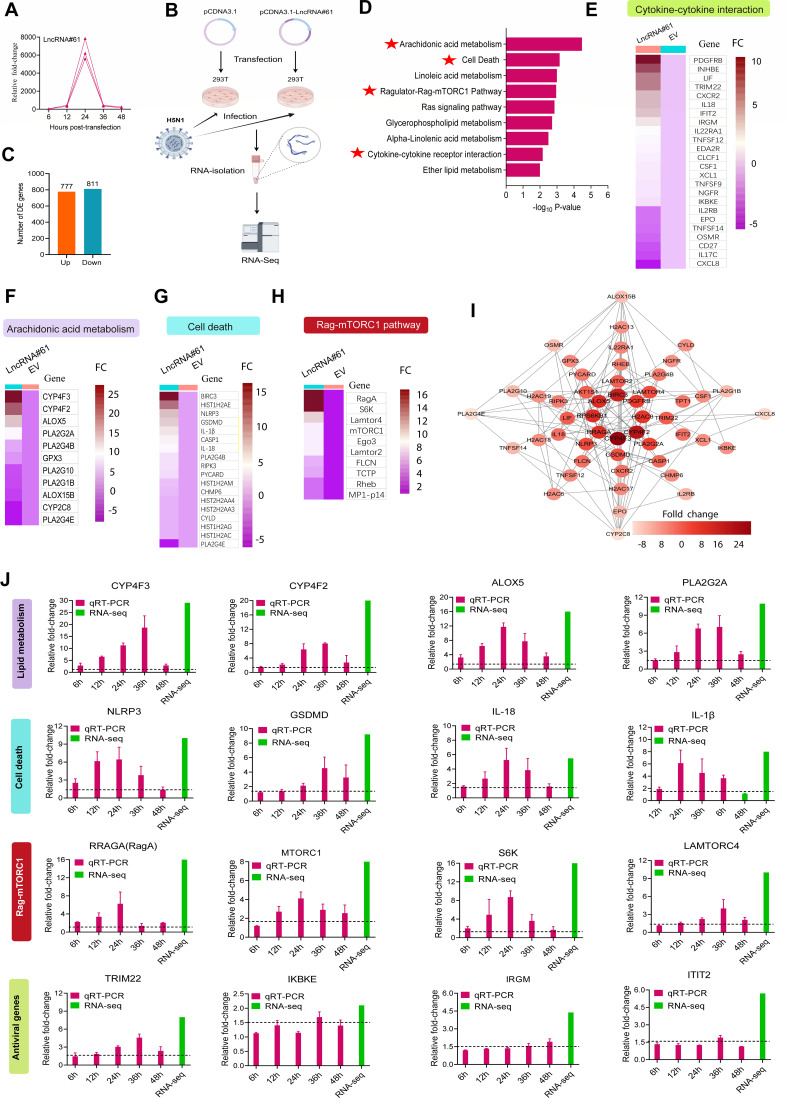
LncRNA#61 enhances lipid metabolism and cell death-related response in 293T cells. (**A**) Relative expression of LncRNA#61 verified by qRT-PCR in 293T cells at different time points after transfection with pcDNA3.1-LncRNA#61 (LncRNA#61). (**B**) Workflow for transcriptome sequencing of 293T cells following a 24-h transfection either with LncRNA#61 or the empty vector (EV) followed by CK10 virus infection. (**C**) Number of SDE genes between LncRNA#61 and EV. (**D**) Top KEGG pathways enriched with the SDE genes. (**E**) Heatmap showing the expression patterns of SDE genes associated with cytokine–cytokine receptor interaction pathway. (**F**) Heatmap showing the expression patterns of SDE genes associated with the arachidonic acid metabolism pathway. (**G**) Heatmap showing the expression patterns of SDE genes associated with the cell death pathway. (**H**) Heatmap showing the expression patterns of SDE genes associated with Ragulator-Rag-mTORC1 signaling pathway. (**I**) Interactome of the SDE genes in panels E through J. Red shading corresponds to fold change identified in RNA-seq data. (**J**) qRT-PCR of the represented genes that screened in RNA-seq data. Data are shown as means ± SD and were compared to the EV group (*n* = 3).

To delineate the antiviral mechanism of LncRNA#61, LncRNA#61 was forcibly expressed in 293T cells, and its expression profile was assessed at multiple time points. Efficient overexpression of LncRNA#61 at 24 h post-transfection was verified by qRT-PCR (Fig. 2A). Following this validation, the global host transcriptional response to LncRNA#61 was analyzed via RNA sequencing at the 24-h time point (Fig. 2B). Upon viral infection, overexpression of LncRNA#61 induced 1,588 significantly differentially expressed (SDE) genes compared to the empty vector (EV) control, comprising 777 upregulated and 811 downregulated SDE genes (Fig. 2C). KEGG pathway enrichment highlighted a significant influence of LncRNA#61 on lipid metabolism and cell death-related pathways, including arachidonic acid (ARA) metabolism, cell death, Ragulator-Rag-mTORC1 signaling, and cytokine–cytokine receptor interaction (Fig. 2D), which was reflected in pronounced expression changes in the associated SDE genes (Fig. 2E through H). A functional interaction network constructed from 63 core genes revealed high functional connectivity among 50 of them (all edges shown, Fig. 2I). Subsequent qRT-PCR validation demonstrated that, compared to the EV, LncRNA#61 overexpression significantly regulated key genes involved in lipid metabolism (CYP4F3, CYP4F2, ALOX5, and PLA2G2A), cell death (NLRP3, GSDMD, IL-18, and IL-1β), Ragulator-Rag-mTORC1 signaling pathway (RRAGA, MTORC1, S6K, and LAMTOR4), and antiviral response (TRIM22, IKBKE, IRGM, and IFIT2) (Fig. 2J).

**Fig 3:**
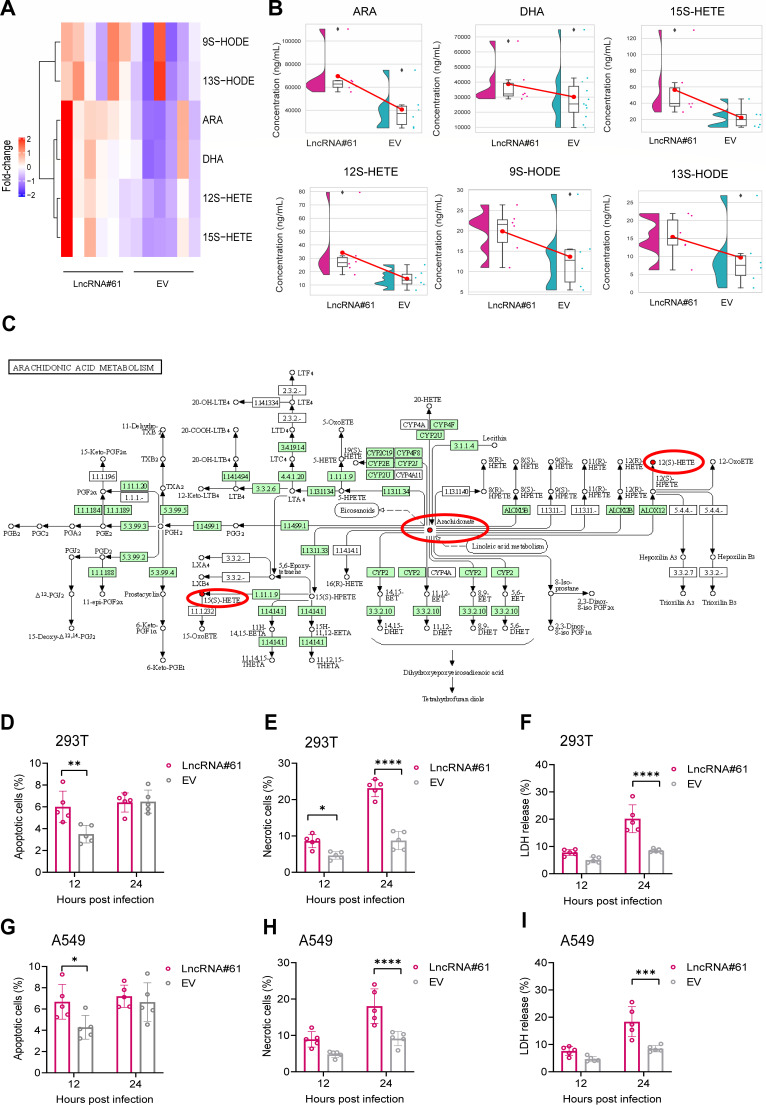
LncRNA#61 enhances arachidonic acid metabolism and cell death in 293T cells. (**A**) Heatmap shows the stimulatory effect of LncRNA#61 on cellular ARA metabolism using targeted metabolomics. (**B**) LncRNA#61 enhances the expression of the metabolites in the cellular ARA metabolism pathway. Data are shown as means ± SD (*n* = 6). (**C**) Metabolites that are significantly upregulated by LncRNA#61 are highlighted in red in the ARA metabolic pathway. (**D–I**) 293 T or A549 cells were transfected either with LncRNA#61 or EV, then infected with the CK10 virus at a multiplicity of infection (MOI) of 2. Data are shown as means ± SD (*n* = 5). (**D and E**) Flow cytometry results for annexin V single positive (**D**) and annexin V and PI dye double positive (**E**) cells at 12 h p.i. or 24 h p.i. in 293T cells. (**F**) LDH release from 293 T cells at 12 h and 24 h p.i. (**G–I**) Flow cytometry results for annexin V single positive (**G**) and annexin V and PI dye double positive (**H**) cells at 12 h p.i. or 24 h p.i. in A549 cells. (**I**) LDH release from A549 cells at 12 h and 24 h p.i. All data are shown as means ± SD and analyzed by two-way ANOVA with Tukey’s multiple comparison test, *P < 0.05, **P < 0.01, ***P < 0.001, ****P < 0.0001.

The transcriptomic results indicate that LncRNA#61 significantly influences lipid metabolism pathways. Recognizing that cell death is often intertwined with cellular metabolic states, we therefore systematically analyzed the influence of LncRNA#61 on cellular metabolism using targeted metabolomics under the condition of viral infection. The results showed that LncRNA#61 significantly upregulated multiple metabolites, including 9S-HODE, 12S-HETE, 13S-HODE, 15S-HETE, ARA, and DHA (Fig. 3A and B; Excel S1). In addition, some of the key metabolites (12S-HETE, ARA, and 15S-HETE) were highly activated in the arachidonic acid metabolism pathway (Fig. 3C). Together with the RNA-seq data (Fig. 2), these findings demonstrate that LncRNA#61 profoundly influences lipid metabolism. Considering the role of LncRNA#61 in regulating cell death and lipid metabolism-related host response, and the considerable role of lipids in regulating cell death (42, 43), we then subsequently assessed the impact of LncRNA#61 on cell death in 293T and A549 cells. At 12 h p.i., LncRNA#61 significantly increased apoptosis in both 293T and A549 cells compared with EV control (Fig. 3D and G). By 24 h p.i., it also markedly elevated necrosis in both cell lines (Fig. 3E and H). Furthermore, lactate dehydrogenase (LDH) release from these cells was also markedly enhanced by LncRNA#61 at 24 h p.i. (Fig. 3F and I). Collectively, these findings indicate that LncRNA#61 orchestrates a coordinated cell death and metabolic response to H5N1 virus infection and is involved in promoting cell death both in 293T and A549 cells.

### LncRNA#61 promotes pyroptosis in LET-1, MDCK, and A549 cells

**Fig 4:**
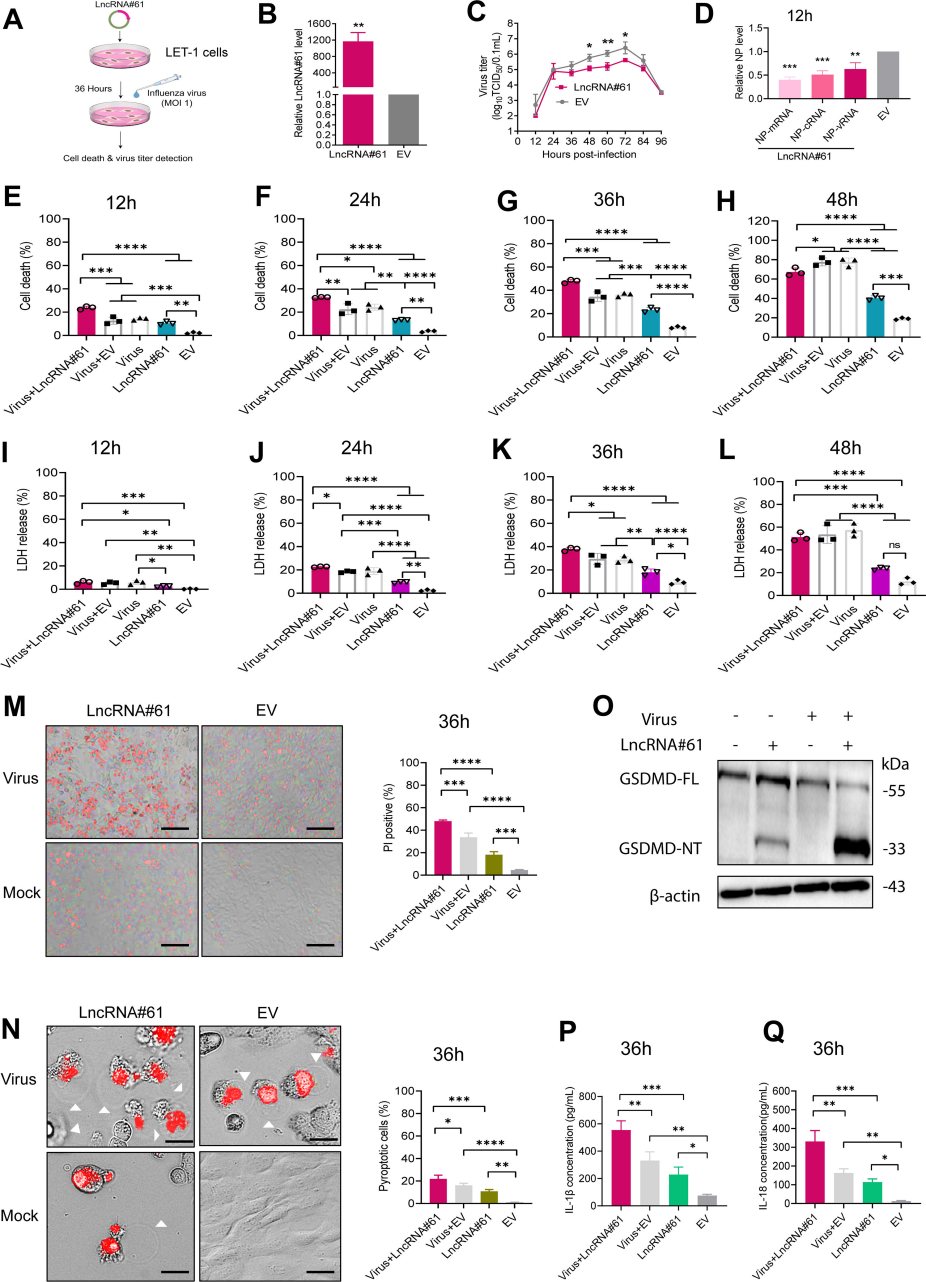
LncRNA#61 promotes pyroptosis in LET-1 cells. (**A**) Schematic diagram of cell death and viral titer detection following forced expression of LncRNA#61 and followed by CK10 virus infection. (**B**) Relative expression of LncRNA#61 in LET-1 cells following transfection with LncRNA#61 for 36 h. (**C and D**) Effects of forced LncRNA#61 expression on viral titers (**C**) and NP mRNA, NP cRNA, and NP vRNA levels (**D**) in LET-1 cells. LET-1 cells were transfected with LncRNA#61 for 36 h, followed by infection with the CK10 virus at an MOI of 0.01. (**E–Q**) Cell death in LET-1 cells. Cells were transfected either with LncRNA#61 or EV for 36 h, followed by infection with CK10 virus at an MOI of 1 or uninfected. Cell death was measured at 12 h (**E**), 24 h (**F**), 36 h (**G**), and 48 h (**H**) p.i. (**I–L**) LDH release was measured as shown in panels E through H at 12 h (**I**), 24 h (**J**), 36 h (**K**), and 48 h (**L**) p.i. (**M**) Cell death image assessed by PI staining (red) at 36 h p.i. Scale bar: 50 µm. Quantitative measurement of PI fluorescence using a plate reader. (**N**) Pyroptosis was assessed by photographing the bubbling cells (indicated by a white triangle) at 36 h p.i. Quantitative measurement of pyroptosis was performed by counting a total of 100 areas. Red staining is represented as positive for PI dye. Scale bar: 10 µm. (**O**) Western blot analysis of the GSDMD expression in LET-1 cells at 36 h p.i. (**P**) IL-1β expression at 36 h p.i. (**Q**) IL-18 expression at 36 h p.i. All data are shown as means ± SD and analyzed by unpaired, two-tailed Student’s t-test (panels B through D) or one-way ANOVA with Tukey’s multiple comparison test (panels E through Q), *P < 0.05, **P < 0.01, ***P < 0.001, ****P < 0.0001.

We previously verified the anti-influenza activity of LncRNA#61 in 293T cells (38). To further assess the potential mechanism underlying its antiviral role, we overexpressed LncRNA#61 in murine LET-1 cells, canine MDCK cells, and human A549 cells (Fig. 4A; Fig. S1A and S2A). qRT-PCR analysis confirmed the successful expression of LncRNA#61 in these cell lines (Fig. 4B; Fig. S1B and S2B). Furthermore, forced expression of LncRNA#61 significantly attenuated viral replication in LET-1 cells (Fig. 4C and D), MDCK cells (Fig. S1C and D), and A549 cells (Fig. S2C and D). Given the established functions of GSDMD, Regulator-RagA-mTORC1 pathway, and lipid metabolism in regulating pyroptosis (43–45), and the strong activation of these pathways by LncRNA#61, we hypothesized that LncRNA#61 might influence pyroptosis during viral infection. This led us to investigate this possibility. Notably, LncRNA#61 markedly accelerated cell death across multiple time points in LET-1, MDCK, and A549 cells, with or without H5N1 infection. This time-dependent effect was evident from 12 h to 48 h in LET-1 cells (Fig. 4E through H), from 12 h to 36 h in MDCK cells (Fig. S1E through H), and at 36 h in A549 cells (Fig. S2E). Consistent with this, LncRNA#61 significantly increased LDH release from LET-1 cells at 24 h and 36 h (Fig. 4I through L), MDCK cells (Fig. S1I through L) at 24 h and 36 h, and A549 cells at 36 h (Fig. S2F).

To further characterize the cell death type induced by LncRNA#61, we performed PI staining and combined it with morphological analysis. At 36 h, regardless of virus infection, LncRNA#61 significantly increased the percentage of PI-positive cells (Fig. 4M; Fig. S1M, O, S2I and K), as well as the proportion of cells exhibiting the balloon-like “bubbling” morphology typical of pyroptotic swelling (Fig. 4N; Fig. S1N, P, S2J and L). This was further supported by the enhanced cleavage of GSDMD by LncRNA#61, the executioner of pyroptosis, as shown in western blot analysis (Fig. 4O). Finally, LncRNA#61 also significantly promoted the release of the key pyroptosis-associated inflammatory mediators, including IL-1β and IL-18 (46), in these cell lines (Fig. 4P and Q; Fig. S1Q through T, S2G and H). Altogether, these data establish that LncRNA#61 potentiates pyroptosis in LET-1 cells, MDCK, and A549 cells, with or without H5N1 influenza virus infection.

### LncRNA#61 enhances GSDMD-mediated pyroptosis through activating Ragulator-RagA

**Fig 5:**
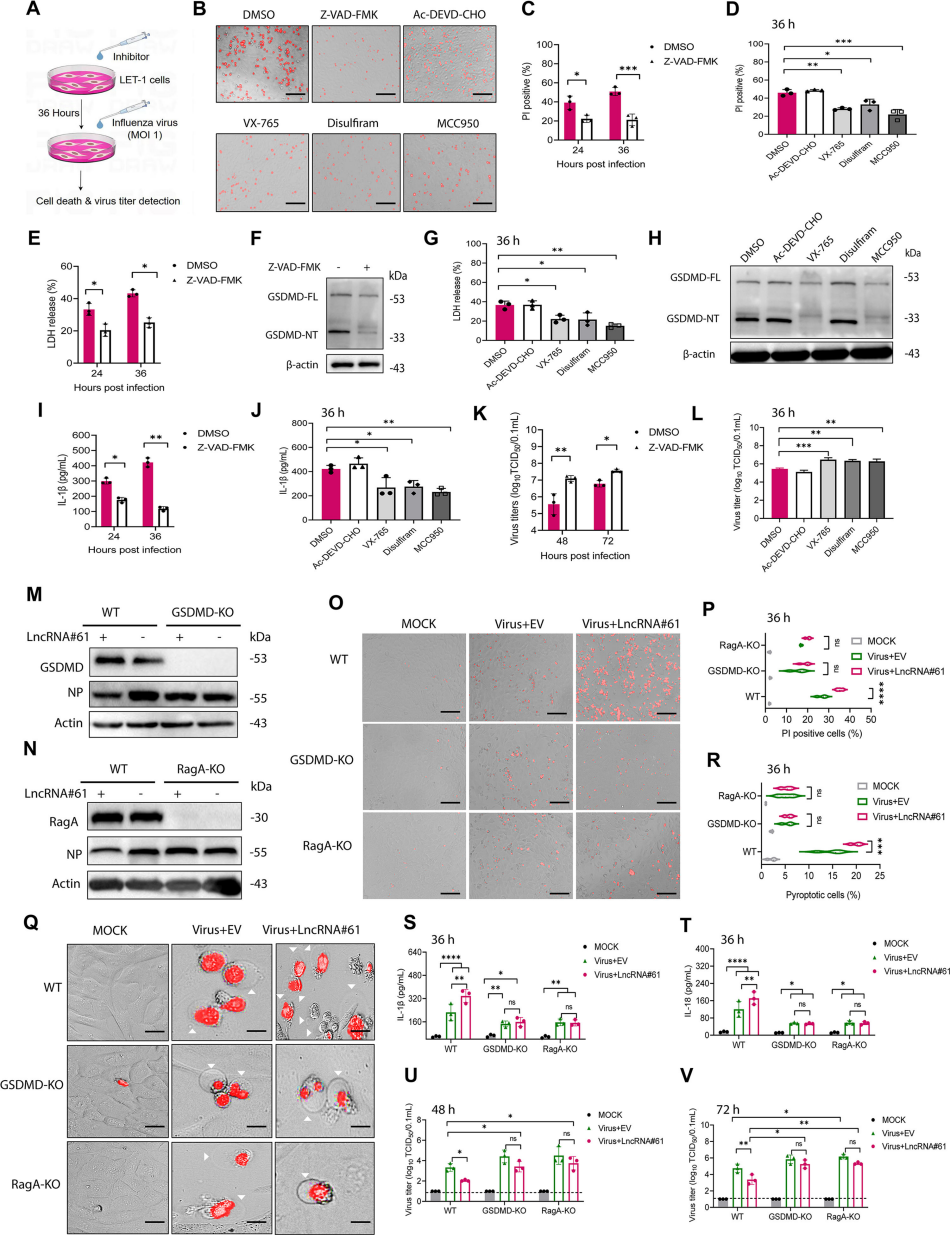
LncRNA#61 enhances GSDMD-mediated pyroptosis via activating RagA in LET-1 cells. (**A**) Schematic diagram of cell death and viral titer detection following pyroptosis inhibitor treatment during forced expression of LncRNA#61 combined with virus infection. (**B**) Cell death image assessed by PI staining after DMSO, Z-VAD-FMK (pan-caspase inhibitor), Ac-DEVD-CHO (Caspase-3 inhibitor), VX-765 (Caspase-1 inhibitor), disulfiram (GSDMD pore formation inhibitor), or MCC950 (NLRP3 inhibitor) treatment, respectively. Scale bar: 50 µm. (**C-D**) Quantitative measurement of PI fluorescence in panel B using a plate reader. (**E**) LDH release of the cells treated with Z-VAD-FMK at 24 h and 36 h p.i. (**F**) Western blot analysis of GSDMD expression in LET-1 cells after treatment with Z-VAD-FMK at 36 h p.i. (**G**) LDH release of the cells treated with Ac-DEVD-CHO, VX-765, disulfiram, or MCC950 at 36 h p.i. (**H**) Western blot analysis of GSDMD expression in LET-1 cells after treatment with Ac-DEVD-CHO, VX-765, disulfiram, or MCC950 at 36 h p.i. (**I**) IL-1β expression of the cells treated with Z-VAD-FMK at 24 h and 36 h p.i. (**J**) IL-1β expression of the cells treated with Ac-DEVD-CHO, VX-765, disulfiram, or MCC950 at 36 h p.i. (**K**) Virus titers of the cells treated with Z-VAD-FMK at 48 h and 72 h p.i. (**L**) Virus titers of the cells treated with VX-765, MCC950, Ac-DEVD-CHO, or disulfiram at 36 h p.i. (**M**) Western blot analysis of GSDMD and viral NP expression in wild-type (WT) LET-1 cells or GSDMD-knockout LET-1 cells at 36 h p.i. (**N**) Western blot analysis of RagA and viral NP expression in wild-type LET-1 cells or RagA-knockout LET-1 cells at 36 h p.i. (**O–V**) Cell death and viral replication in wild-type LET-1 cells, GSDMD, or RagA-knockout LET-1 cells that were transfected either with LncRNA#61 or EV, followed by virus infection or mock infection. (**O**) Cell death image was assessed by PI staining (red) at 36 h p.i. Scale bar: 50 µm. (**P**) Quantitative measurement of PI fluorescence in panel O using a plate reader. (**Q**) Pyroptosis was assessed by photographing the bubbling cells (indicated by a white triangle) at 36 h p.i. Red staining is represented as positive for PI dye. Scale bar: 10 µm. (**R**) Quantitative measurement of pyroptosis in panel Q. (**S**) IL-1β expression at 36 h p.i. (**T**) IL-18 expression at 36 h p.i. (**U**) Viral replication at 48 h p.i. (**V**) Viral replication at 72 h p.i. All data are shown as means ± SD and analyzed by one-way (panels D, G, J, and L) or two-way ANOVA with Tukey’s multiple comparison test (panels C, E, I, K, and P through V), *P < 0.05, **P < 0.01, ***P < 0.001, ****P < 0.0001.

To further investigate the mechanism by which LncRNA#61 induces pyroptosis, we performed pyroptosis pathway inhibition assays in LET-1 cells using five specific inhibitors: Z-VAD-FMK (a pan-caspase inhibitor targeting caspases 1, 3, and 8), VX-765 (a caspase-1 inhibitor), Ac-DEVD-CHO (a caspase-3 inhibitor), MCC950 (an NLRP3 inhibitor), and disulfiram (a GSDMD inhibitor that directly inhibits GSDMD pore formation activity). We first assessed the effect of these inhibitors on cell death by PI staining of the LET-1 cells transfected with LncRNA#61, followed by virus infection (Fig. 5A). As shown in Fig. 5B through D, the number of PI-positive cells was significantly reduced in cell cultures treated with Z-VAD-FMK, VX-765, disulfiram, or MCC950 compared with the DMSO control, but not with Ac-DEVD-CHO. Correspondingly, LDH release was also attenuated in LET-1 cells treated with these inhibitors (Fig. 5E and G). Furthermore, immunoblot analysis revealed that Z-VAD-FMK, VX-765, and MCC950 each suppressed GSDMD cleavage, whereas Ac-DEVD-CHO and disulfiram showed no significant effect (Fig. 5F and H). Since IL-1β is processed by inflammatory caspases and released through pores formed by cleaved GSDMD, we measured its secretion under inhibitor treatment. Concentrations of IL-1β were markedly lower in cells exposed to Z-VAD-FMK, VX-765, disulfiram, or MCC950 (Fig. 5I and J). Next, to examine the role of pyroptosis in viral replication, we measured viral titers following treatment with these inhibitors. We observed that inhibitors which suppress pyroptosis consistently increased viral titers (Fig. 5K and L). Altogether, these findings indicate that GSDMD contributes to trigger LncRNA#61 and virus infection-mediated pyroptosis, and that pyroptosis is negatively correlated with viral replication.

Based on the RNA-seq analysis, forced expression of LncRNA#61 significantly upregulated genes associated with the Ragulator-Rag-mTORC1 pathway, including RRAGA (hereafter referred to as RagA), MTORC1, S6K, LAMTOR2, and LAMTOR4 (Fig. 2H through J). RagA acts as a key amino acid-sensing “molecular switch” within this pathway, cycling between GTP- and GDP-bound states to regulate mTORC1 localization and activity. Given this central role and the established role of the Ragulator-Rag-mTORC1 pathway in promoting GSDMD oligomerization, pore formation, and pyroptosis (44), we then sought to investigate the roles of GSDMD and RagA in LncRNA#61-regulated pyroptosis. To this end, we utilized GSDMD- and RagA-knockout LET-1 cells. Western blot analysis confirmed the successful knockout of GSDMD and RagA in LET-1 cells (Fig. 5M and N). Correspondingly, the ability of LncRNA#61 to enhance cell death, as measured by PI staining, was observed in wild-type (WT) cells but was abolished in both GSDMD- and RagA-knockout cells (Fig. 5O and P). We next tested whether GSDMD- or RagA-knockout affected pyroptosis. Similarly, LncRNA#61’s promotion of pyroptosis and the subsequent release of IL-1β and IL-18 were strictly dependent on GSDMD and RagA, as no increase was seen in these knockout cells (Fig. 5Q through T). Interestingly, forced expression of LncRNA#61 significantly decreased viral load at 48 h and 72 h p.i. in wild-type LET-1 cells, while this advantage was not observed both in GSDMD- or RagA-knockout LET-1 cells (Fig. 5U and V). In addition, virus titers in the knockout cells were higher than in WT cells. Collectively, these results clearly indicate that GSDMD-mediated pyroptosis restricts viral replication in LET-1 cells, and that LncRNA#61 enhances this pathway by activating the RagA-GSDMD axis.

### LncRNA#61-PA-X interaction promotes pyroptosis via RagA-dependent ROS pathway

**Fig 6:**
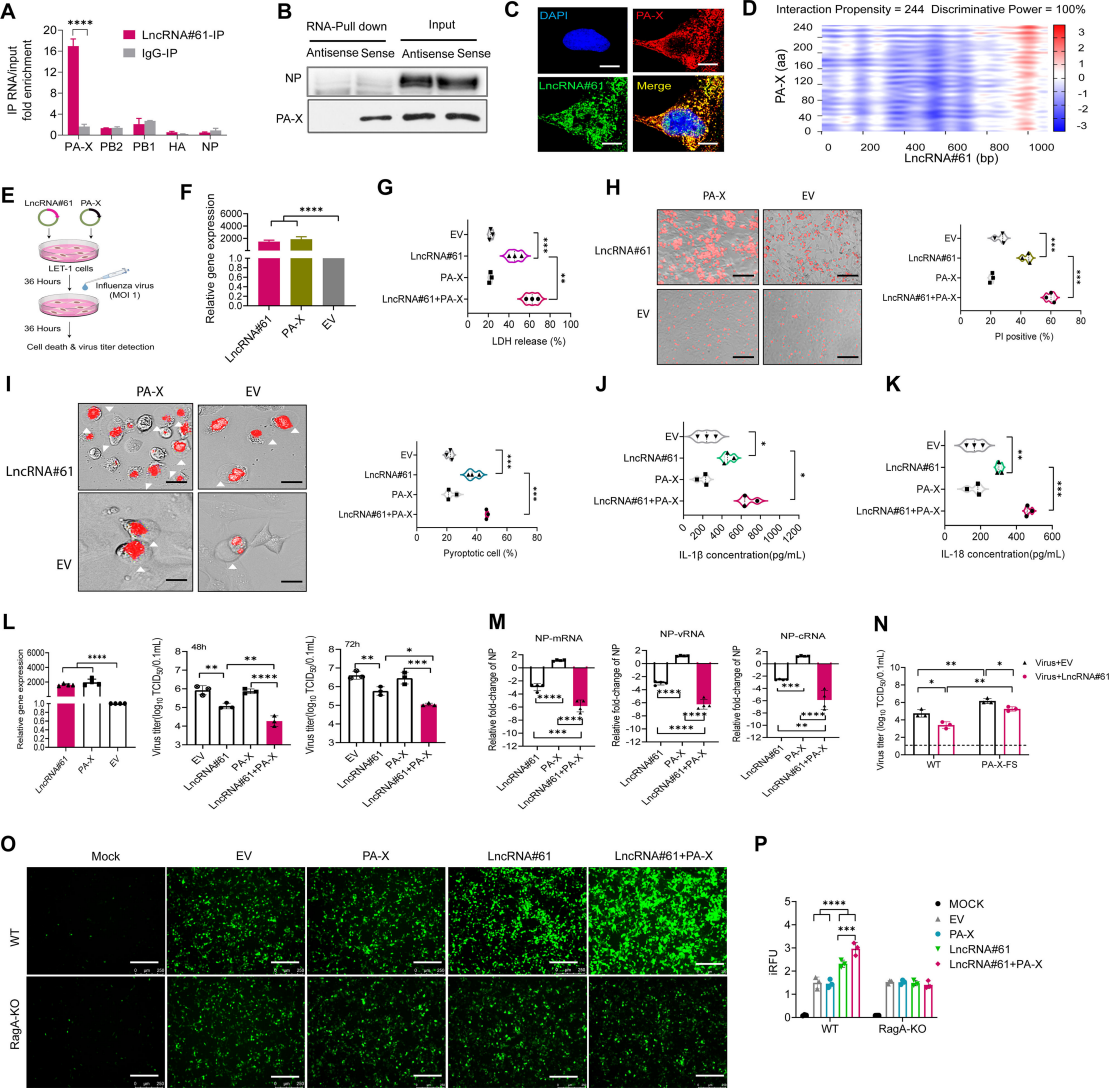
LncRNA#61-PA-X interaction promotes pyroptosis through RagA-dependent ROS pathway in LET-1 cells. (**A**) Cell lysates were subjected to immunoprecipitation using antibodies against PB2, PB1, NP, HA, or PA-X, with normal IgG as a control. The co-precipitated RNA was then analyzed by qRT-qPCR to detect the amount of LncRNA#61, with β-actin mRNA as an internal reference. (**B**) The LncRNA#61 pull-down proteins were detected by immunoblotting using anti-PA-X or -NP antibody. (**C**) LET-1 cells were fixed and processed for RNA FISH using a biotin-labeled antisense probe specific to LncRNA#61 (green), followed by immunostaining with an anti-PA-X primary antibody (red). Nuclei were counterstained with DAPI (blue). Scale bar, 5 μm. (**D**) catRAPID analysis (http://service.tartaglialab.com) indicated a strong interaction between LncRNA#61 and PA-X. (**E**) Schematic diagram of cell death and viral titer detection following forced expression of LncRNA#61 and PA-X, and followed by CK10 virus infection. (**F**) Relative expression level of LncRNA#61 and PA-X was determined by qRT-qPCR. (**G**) LDH release of the LET-1 cells at 36 h p.i. (**H**) PI staining (red) images of the LET-1 at 36 h p.i. Scale bar: 50 µm. Quantitative measurement of PI fluorescence using a plate reader. (**I**) Pyroptosis was assessed by photographing of the bubbling cells (indicated by a white triangle) at 36 h p.i. Quantitative measurement of pyroptosis was performed by counting a total of 100 areas. Red staining is represented as positive for PI dye. Scale bar: 10 µm. (**J**) IL-1β expression at 36 h p.i. (**K**) IL-18 expression at 36 h p.i. (**L–M**) Effect of overexpression of LncRNA#61 and PA-X on viral titers (**L**) and viral NP mRNA, NP cRNA, and NP vRNA (**M**) at 48 h and 72 h p.i. (**N**) Virus titers in LET-1 cells transfected with LncRNA#61 or EV followed by infection either with the wild-type H5N1 virus (WT) or the PA-X-deficient virus (PA-X-FS). (**O**) Wild-type or RagA-knockout LET-1 cells were transfected with the indicated plasmids. At 36 h post-transfection, cells were infected with CK10 virus at an MOI of 1. After a further 24 h, cells were incubated with 10 μM DCFH-DA for fluorescence observation. Scale bar: 250 µm. (**P**) Cells treated as described above were loaded with 10 μM CM-H₂DCFDA, and the fluorescence was immediately measured using a plate reader at 24 h p.i. All data are shown as means ± SD and analyzed by unpaired, two-tailed Student’s *t*-test (panels A and F) or one-way ANOVA with Tukey’s multiple comparison test (panels G through M) or two-way ANOVA with Tukey’s multiple comparison test (panels N and P), *P < 0.05, **P < 0.01, ***P < 0.001, ****P < 0.0001.

To determine whether viral factors modulate the pyroptosis-inducing ability of LncRNA#61, we first screened for its interacting viral partners. RNA immunoprecipitation (RIP) identified the viral protein PA-X as a candidate (Fig. 6A). This interaction was further validated by an RNA pull-down assay using biotin-labeled LncRNA#61 (Fig. 6B). Notably, to substantiate the direct binding, we performed RNA fluorescence *in situ* hybridization (RNA-FISH) for LncRNA#61 combined with immunofluorescence assay (IFA) for PA-X in MDCK cells, which revealed clear co-localization of LncRNA#61 and PA-X (Fig. 6C). In addition, computational prediction using the catRAPID algorithm supported a high binding propensity between LncRNA#61 and PA-X (Fig. 6D) (47). Collectively, these results revealed the direct interaction of LncRNA#61 and PA-X. To address whether PA-X influences LncRNA#61-mediated pyroptosis, we co-expressed both factors in LET-1 (experimental schemes in Fig. 6E; expression verified by qRT-PCR in Fig. 6F) and MDCK cells (experimental schemes in Fig. S3A; expression verified by qRT-PCR in Fig. S3B). Following H5N1 infection, the co-expression group exhibited markedly higher LDH release than groups expressing either factor alone (Fig. 6G; Fig. S3C), whereas PA-X alone had no effect in this aspect. Correspondingly, PI staining revealed a significant increase in PI-positive cells upon co-expression in both cell types (Fig. 6H; Fig. S3D). Similarly, the proportion of morphologically defined pyroptotic cells was substantially elevated in the co-expression groups (Fig. 6I; Fig. S3E). In line with these observations, co-expression of LncRNA#61 and PA-X led to a significant increase in secretion of IL-1β and IL-18 compared to LncRNA#61 alone, in both LET-1 (Fig. 6J and K) and MDCK cells (Fig. S3F and G). Most importantly, this synergistic interaction resulted in a significantly greater suppression of viral replication in LET-1 cells (Fig. 6L and M) and MDCK cells (Fig. S3H) than either factor alone.

To investigate whether the observed effect depends on the viral expression of PA-X, we further compared the role of LncRNA#61 in regulating viral replication of the WT virus and the PA-X-deficient virus (PA-X-FS). As a result, when infected with the WT virus, compared with EV, forced expression of LncRNA#61 significantly decreased viral titers of the WT virus (Fig. 6N). When infected with the PA-X-FS, compared with EV, LncRNA#61 again significantly decreased viral titers of the PA-X-deficient virus. Meanwhile, PA-X-FS replicated more efficiently both in EV- and LncRNA#61-treated cells than that of the corresponding WT virus. Therefore, these results demonstrate that LncRNA#61 inhibits viral replication independent of PA-X protein. Moreover, in a virus context (not PA-X protein alone), PA-X significantly inhibits viral replication during forced expression of LncRNA#61 or not. We also demonstrated that the synergy was specific to PA-X because forced expression of other viral proteins (such as HA or PB1) failed to augment LncRNA#61-mediated phenotypes, showing no significant effect on cell-death-related gene expression (Fig. S4A through C; Fig. S5A through C) or on viral replication in LET-1 cells (Fig. S4D through F; Fig. S5D through F).

Mitochondrial dysfunction, which is linked to both GSDMD pore formation and Ragulator-Rag activity (44), often coincides with increased cellular reactive oxygen species (ROS), a known promoter of GSDMD pore formation (44, 48). Given this link, we next asked whether LncRNA#61-PA-X interaction affects ROS production. Using a commercial ROS assay kit, the LncRNA#61-PA-X interaction was found to significantly elevate ROS levels in WT LET-1 cells, but not in RagA-deficient LET-1 cells (Fig. 6O). Consistently, CM-H2DCFDA fluorescence staining showed a markedly higher increase in ROS intensity (iRFU) only in WT LET-1 cells upon co-expression of LncRNA#61 and PA-X (Fig. 6P). Thus, these data identify RagA as a critical mediator of the ROS induction linked to GSDMD pore formation activity. Collectively, our findings demonstrate that the LncRNA#61-PA-X interaction enhances pyroptosis through a RagA-ROS-GSDMD axis, thereby attenuating viral replication in LET-1, A549, and MDCK cells.

### LncRNA#61-PA-X interaction boosts pyroptosis in mouse lung

**Fig 7:**
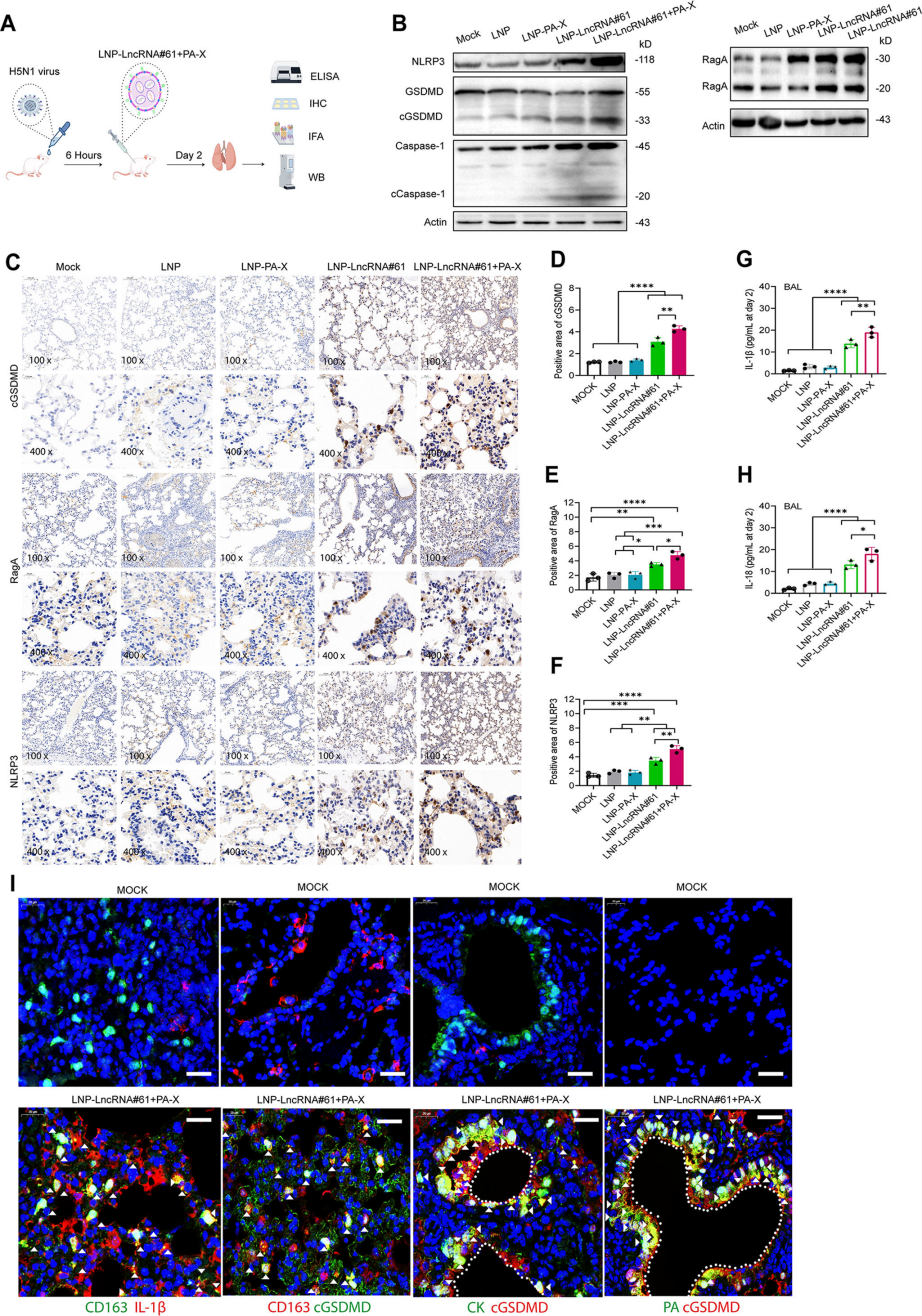
LncRNA#61-PA-X interaction boosts pyroptosis in mouse lung. (**A**) Schematic diagram of the experiment investigating cell-death-related protein expression in mouse lung following LNP-mediated overexpression of LncRNA#61 and PA-X (mRNA) and subsequent CK10 virus infection. (**B**) Western blot analysis of NLRP3, GSDMD, Caspase-1, and RagA expression in mouse lung at days 2 p.i. (**C**) IHC analysis of the expression of the cleaved GSDMD (cGSDMD), RagA, and NLRP3 in mouse lung at days 2 p.i. (**D–F**) Quantitative analysis of pyroptosis-related proteins in mouse lung in panel C. (**D**) cGSDMD. (**E**) RagA. (**F**) NLRP3. (**G**) The concentration of IL-1β in BALF at 2 days p.i. in mice. (**H**) The concentration of IL-18 in BAL fluid at 2 days p.i. in mice. (**I**) Confocal images of lung sections from untreated mice and mice subjected to LNP-mediated overexpression of LncRNA#61/PA-X combined with viral infection. Sections were co-stained with antibodies against CD163, cytokeratin (CK), viral PA protein, IL-1β, or cGSDMD, together with DAPI (blue). Dotted lines outline alveoli, arrowheads indicate double-labeled (orange) cells. Scale bar: 20 µm. All data are shown as means ± SD and analyzed by one-way ANOVA with Tukey’s multiple comparison test, *P < 0.05, **P < 0.01, ***P < 0.001, ****P < 0.0001.

To evaluate the effect of the LncRNA#61-PA-X interaction on pyroptosis *in vivo*, mice were inoculated with LNP-LncRNA#61-PA-X, LNP-LncRNA#61, LNP-PA-X, or LNP 6 h after H5N1 infection (Fig. 7A). Analysis of lung tissues on day 2 p.i. revealed that LNP-LncRNA#61-PA-X significantly increased the protein levels of the cleaved GSDMD (cGSDMD), NLRP3, cleaved caspase-1 (cCaspase-1), and RagA compared to LNP-LncRNA#61 (Fig. 7B). Consistently, immunohistochemistry (IHC) in mouse lung demonstrated stronger expression of cGSDMD, NLRP3, and RagA in LNP-LncRNA#61-PA-X and LNP-LncRNA#61 groups than that of in controls, with the combination group showing the highest signal (Fig. 7C through F). Furthermore, bronchoalveolar lavage fluid (BALF) from the LNP-LncRNA#61-PA-X group contained significantly higher levels of IL-1β and IL-18 (Fig. 7G and H). Collectively, these data indicate that LncRNA#61-PA-X interaction synergistically promotes pyroptosis in the lung during early viral infection time. To identify the cell types undergoing pyroptosis, we performed IFA for IL-1β and cGSDMD, combined with markers for macrophages (CD163) and alveolar epithelial cells (cytokeratin, CK). While lungs from mock-inoculated mice showed negligible signals, those receiving LNP-LncRNA#61-PA-X exhibited widespread IL-1β expression and cGSDMD (Fig. 7I). Furthermore, cGSDMD was induced and colocalized with both CD163^+^ macrophages and cytokeratin^+^ (CK) alveolar epithelial cells. Strikingly, most cGSDMD+ cells were also positive for the viral PA protein, and these infected, double-positive cells were primarily alveolar-lining cells. Collectively, these findings indicate that, under the condition of ectopic expression of LncRNA#61 and PA-X, LncRNA#61-PA-X interaction enhances pyroptosis in mice by promoting inflammatory caspase activation, GSDMD cleavage, and IL-1β production within infected lung macrophages and alveolar epithelial cells, thereby restraining viral replication.

### LncRNA#61-PA-X interaction has obvious anti-influenza activity in mice

**Fig 8:**
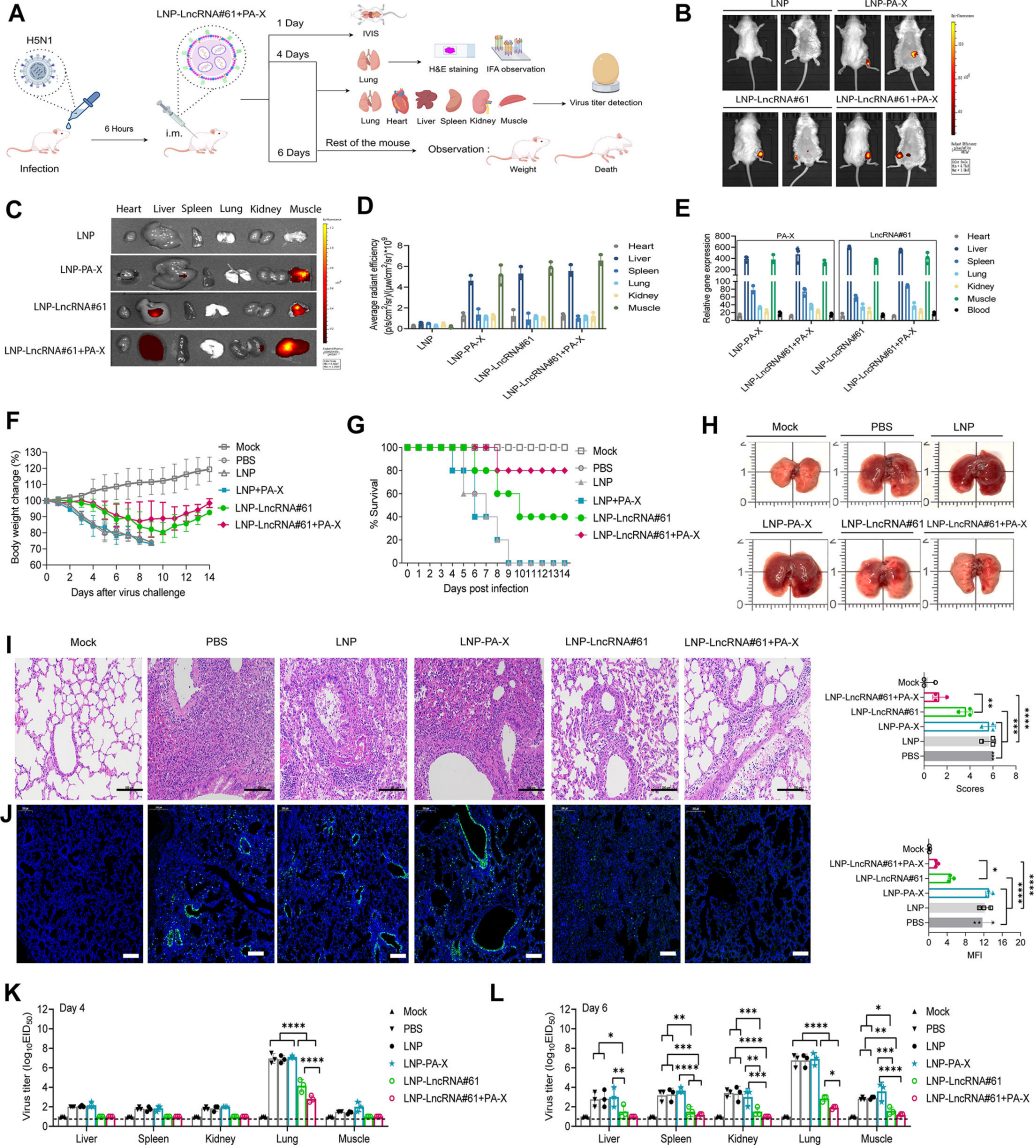
LncRNA#61-PA-X interaction has obvious anti-influenza activity in mice. (**A**) Schematic diagram of the experiment investigating the protective efficacy of the LNP-mediated overexpression of LncRNA#61 and PA-X during lethal H5N1 influenza virus infection. (**B**) *In vivo* distribution of LNP-LncRNA#61 in mice. *In vivo* imaging was performed at 24 h post-inoculation. (**C**) Following imaging, major organs were collected at the corresponding time points for *ex vivo* fluorescence measurement of LncRNA#61 distribution. (**D**) The fluorescence intensity of LncRNA#61 in panel C (*n* = 3). (**E**) Mouse organs were collected at the indicated time points for qRT-PCR analysis of LncRNA#61 (*n* = 3). (**F**) Body weight and (**G**) survival rate of the mice were monitored daily for 14 days (*n* = 5). (**H**) Necropsy was performed for observation of gross lung pathology at 6 days p.i. (**I**) H&E staining and pathological scoring of the lung tissues from different groups of mice at 6 days p.i. Scale bar: 100 µm. (**J**) Immunostaining of lung tissues with an influenza virus NP-specific mAb and quantification of the MFI at 6 days p.i. Scale bar: 200 µm. (**K and L**) Viral replication in mouse organs at days 4 p.i. (**K**) (*n* = 3) and at days 6 p.i. (**L**) (*n* = 3). All data are shown as means ± SD, and analyzed by one-way ANOVA with Tukey’s multiple comparison test (panels I and J) or unpaired and two-tailed Student’s t-test (panels K and L), *P < 0.05, **P < 0.01, ***P < 0.001, ****P < 0.0001.

Based on the finding that forced expression of PA-X enhances LncRNA#61-induced pyroptosis, we next assessed the functional consequences of this interaction on viral infection and disease severity *in viv*o. Following the scheme in Fig. 8A, groups of mice were inoculated with LNP-LncRNA#61-PA-X, LNP-LncRNA#61, LNP-PA-X, LNP, or PBS control 6 h after H5N1 infection. Mice were monitored daily for body weight and survival, and organs were collected for virus titration and pathological examination. *In vivo* imaging at 24 h post-delivery showed that both LncRNA#61 and PA-X were detectable in multiple organs, including muscle, liver, heart, spleen, and kidneys (Fig. 8B through D). qRT-PCR analysis confirmed their successful delivery, revealing significantly higher levels of LncRNA#61 and PA-X in mice inoculated with the respective LNP formulations compared to the LNP-control group (Fig. 8E). Mice treated with LNP-LncRNA#61-PA-X or LNP-LncRNA#61 exhibited attenuated weight loss (Fig. 8F) and an obvious improvement in survival (Fig. 8G), with protection rates of 80% and 40%, respectively. Consistent with these clinical benefits, gross pathological examination at 6 days p.i. revealed severe pulmonary hemorrhage in control (LNP and PBS) and LNP-PA-X groups (Fig. 8H). In contrast, lung damage was markedly reduced in mice receiving LNP-LncRNA#61 or LNP-LncRNA#61-PA-X, with the latter showing the most pronounced protection. Histological assessment at 6 days p.i. showed that lungs from PBS, LNP- or LNP-PA-X-treated mice exhibited severe inflammatory infiltration, alveolar wall congestion, thickening, and hemorrhages (Fig. 8I). These pathological changes were markedly attenuated in the LNP-LncRNA#61 and LNP-LncRNA#61-PA-X groups, with the latter being the most protected. Accordingly, quantitative histopathological scores were significantly lower in the LNP-LncRNA#61-PA-X group (Fig. 8I). Further analysis showed obviously reduced NP protein fluorescence in the lungs of LNP-LncRNA#61-PA-X-treated mice compared to the LNP-LncRNA#61 group (Fig. 8J). Moreover, viral titers in the lung (4 days p.i.) and in multiple organs including the lung, liver, spleen, muscle, and kidneys (6 days p.i.) were significantly lower in mice treated with either LNP-LncRNA#61-PA-X or LNP-LncRNA#61 than in control groups (Fig. 8K and L). Notably, at both time points, the LNP-LncRNA#61-PA-X group exhibited the most potent viral suppression, with significantly lower titers in the lung than those of LNP-LncRNA#61. Taken together, these *in vivo* results demonstrate that, under condition of ectopic expression of PA-X coupled with LncRNA#61, LncRNA#61-PA-X interaction potently suppresses IAV replication and pathogenesis, highlighting its promise as a target for novel antiviral therapies.

## DISCUSSION

The extensive antigenic diversity of IAVs, along with limited cross-protective immunity and the rapid emergence of drug resistance, highlights an urgent need for broad-spectrum antiviral agents that possess a high genetic barrier to resistance (49). Host-targeting antivirals (HTAs) offer a promising strategy to address this challenge (5, 6). Although LncRNAs are increasingly recognized for their roles in viral infection and innate immune regulation (18, 50), their multifaceted functions within immune-metabolic networks during infection remain poorly understood. Our previous study identified LncRNA#61 as a broad-spectrum host factor against influenza virus (38). Here, we elucidate an antiviral mechanism through which LncRNA#61 promotes pyroptosis by activating the RagA-ROS-GSDMD axis. Notably, forced expression of viral protein PA-X acts synergistically with LncRNA#61 to amplify this pyroptotic pathway, providing fresh mechanistic insight into how a host lncRNA and a viral factor cooperate to achieve viral restriction.

Pyroptosis, an inflammatory cell death pathway mediated by gasdermin (GSDM) family pore-forming proteins, can be triggered by various damage signals. In humans, the six known GSDM members (GSDMA-E and PJVK) respond differentially to such signals. Here, we reveal that the long noncoding RNA-LncRNA#61 triggers GSDMD-dependent pyroptosis both in mouse lung epithelial type I (LET-1) cells and *in vivo*. It is well established that IAV infection induces pyroptosis primarily in macrophages and epithelial cells. In mouse bone marrow-derived macrophages (BMDMs), IAV typically activates the NLRP3 inflammasome-caspase-1 axis, leading to GSDMD cleavage and pyroptosis (51). In respiratory mammalian epithelial cells, however, the pathway diverges depending on viral strain and cell type. For example, H5N1 triggers GSDMD-mediated pyroptosis in macaque alveolar epithelial cells (34), whereas H7N9 activates caspase-3 to cleave GSDME in human alveolar cells (33). Similarly, H1N1 strains can engage either caspase-3/GSDME in normal human bronchial epithelial (NHBE) cells (52) or both caspase-8/GSDMD and caspase-3/GSDME in murine LET-1 cells and human bronchial epithelial cells (NL20) (53). Building upon this knowledge, our work extends the understanding that LncRNA#61 promotes H5N1 virus-induced pyroptosis by demonstrating GSDMD-mediated cell death in LET-1 cells, mouse alveolar macrophages, and alveolar epithelial cells. However, to strengthen the physiological relevance, future studies still need to validate these phenotypes in more physiologically relevant models, such as human primary cells or air-liquid interface (ALI) cultures. While the present study demonstrates that LNP-mediated delivery of LncRNA#61 exerts significant antiviral and cell death-promoting effects *in vivo*, it must be acknowledged that all comparisons were made against empty LNPs. This design, while appropriate for establishing a delivery-based therapeutic effect, does not formally exclude the possibility that introducing other large or structured RNAs might also produce similar outcomes. Our previous work with truncation mutants of LncRNA#61 indicated that its function required specific domains, not merely transcript length, as certain large truncations lacking these domains were inactive (38). However, the longest truncation mutant retained activity, suggesting that a minimum structural framework or length may be necessary. Therefore, to unequivocally establish the sequence specificity of LncRNA#61, future studies should include controls such as a scrambled RNA sequence or a distinct, unrelated lncRNA delivered via the same LNP platform. This important caveat highlights a key direction for further mechanistic validation.

Our present study demonstrates that LncRNA#61 enhances pyroptosis to restrict H5N1 replication, thereby mitigating lung pathology in mice. The pyroptosis process, however, is a double-edged sword during virus infection. In the lower respiratory tract, the magnitude of pyroptosis critically dictates the balance between pathogen clearance and immunopathology. Moderate pyroptosis aids defense, whereas excessive activation drives deleterious inflammation, tissue damage, and death (33, 54–56). For instance, while lung epithelial pyroptosis protects against melioidosis (46), H7N9-induced pyroptosis triggers a harmful cytokine storm, and GSDME knockout improves survival of the mice (33). Similarly, SARS-CoV-2-induced pyroptosis curbs viral replication but also contributes to COVID-19 hyperinflammation (54). Strikingly, we found that the viral PA-X protein amplifies LncRNA#61-mediated pyroptosis, leading to a protective outcome that reduces H5N1 pathogenesis. It is generally accepted that type I and type II alveolar epithelial cells (AECs) represent major targets and replication sites for IAV in the lower respiratory tract (57–59). Loss of more than ∼10% of type I AECs strongly predicts mortality (60). This lethal threshold also encompasses bystander AECs-uninfected cells that are eliminated by immune effectors such as CD8^+^ T cells within the inflamed tissue milieu (61). Although type II AECs can act as progenitors for lung repair by differentiating into type I AECs, impaired or incomplete differentiation into functional alveolar epithelium results in maladaptive repair and persistent pulmonary pathology (62, 63). Thus, a finely balanced cell death response is essential to reconcile immune-mediated protection with immunopathology, an equilibrium that ultimately defines the benefit of the antiviral response. However, the precise mechanism by which LncRNA#61 synergistically interacts with PA-X to amplify pyroptosis and finally protect the host from injury remains to be further explored. Moreover, while the current study employed systemic delivery, future work could explore the localized administration of LNP-LncRNA#61 + PA-X (e.g., via intranasal or pulmonary routes) to the respiratory tract. It is plausible that such a strategy could enhance target tissue concentration while potentially mitigating risks of systemic inflammation.

Excessive pyroptosis and necroptosis can drive harmful cytokine storms (64), prompting interest in inflammasome inhibition for treating viral hyperinflammation. However, the inflammasome acts as a two-edged blade, playing roles in both protective antiviral defense (65–67) and pathological inflammation (68–70). This is illustrated by the increased susceptibility of NLRP3-deficient mice to influenza (65, 66) and relatively lower cytokine levels in some severe COVID-19 cases compared to sepsis (71). Therefore, any therapeutic intervention must be carefully tailored to the specific disease stage and pathological context. A major challenge in managing pyroptosis-related immunopathology is to identify regulators that can restrain excessive inflammation while preserving essential antiviral defenses. In this context, we observed that LncRNA#61 not only promotes pyroptosis but also upregulates the expression of several antiviral genes (e.g., TRIM22, IKBKE, IRGM, and IFIT2) (Fig. 2I). This dual activity suggests that LncRNA#61’s pro-pyroptotic role may be complemented by a broader transcriptional program, collectively enhancing the overall antiviral response. However, a key remaining question for future studies is the precise mechanism by which LncRNA#61 upregulates these genes. Interestingly, our previous study revealed that LncRNA#61 inhibits viral polymerase (38). Here, we identified induction of pyroptosis as another mechanism by which LncRNA#61 exerts its antiviral function. Although these two functions appear distinct, together they constitute a coordinated host defense system. We speculate that the LncRNA#61-mediated polymerase inhibition may serve as an upstream triggering event. On one hand, it directly limits the efficiency of viral genome replication. On the other hand, by blocking viral transcription, it may cause aberrant viral products to accumulate, which enhances pattern recognition receptor signaling and ultimately drives inflammasome activation and pyroptosis. Furthermore, these two functions may represent temporally and spatially distinct layers of antiviral defense: early in infection, LncRNA#61 maintains homeostasis by inhibiting viral polymerase; once viral replication exceeds a threshold, it eliminates the infection “factory” via pyroptosis. Although other biological functions of LncRNA#61 remain unclear, our data suggest that it plays a critical role as a pleiotropic regulator in antiviral immunity.

Pyroptosis plays complex and often paradoxical roles during viral infections, and its specific function in regulating influenza virus replication remains incompletely defined. This complexity is evident in conflicting reports. On one hand, caspase-6 promotes IAV-induced PANoptosis (pyroptosis, apoptosis, and necroptosis) to restrain infection and pathology in PR8 (H1N1)-infected mice, although the specific contribution of pyroptosis remains unclear (32). Conversely, ZBP1 deficiency, which prevents pyroptosis and increases viral replication, still fails to protect mice from lethal H1N1 infection (58). In this study, we directly demonstrated that pyroptosis acts to inhibit H5N1 viral replication both *in vitro* and *in vivo*. To counteract pyroptosis, viruses have evolved diverse molecular strategies. For instance, the PB1-F2 protein of contemporary H5N1 and H3N2 viruses suppresses pyroptosis by limiting NLRP3-NEK7 complex assembly in human macrophages (72). SARS-CoV-2 nucleocapsid protein inhibits pyroptosis via directly binding to GSDMD and preventing its cleavage by caspase-1 (73). Similarly, EV71 3C protease inactivates GSDMD by cleaving it at Q193-G194, resulting in a truncated N-terminal fragment that lacks pore-forming activity (74). In contrast, we here discovered that, unlike typical viral evasion tactics, the PA-X protein enhances LncRNA#61-mediated pyroptosis, leading to a restraint of H5N1 viral infection and pathogenesis in mice. Notably, the influenza virulence factor PB1-F2 also exacerbates pathological injury by activating the NLRP3 inflammasome (75). Likewise, the G-protein of spring viremia of carp virus (SVCV) activates NLRP3, driving inflammation and pyroptosis that lead to tissue necrosis and hemorrhages (76). Therefore, these examples collectively demonstrate how diverse viruses co-opt inflammasome signaling to facilitate infection or worsen immunopathology.

We previously reported that PA-X attenuates H5N1 pathogenicity by inhibiting viral replication and host immune responses (77, 78), we here extend this understanding by showing that forced expression of PA-X fine-tunes LncRNA#61-mediated pyroptosis to restrain viral infection. We propose a new model in which LncRNA#61 and PA-X interaction optimizes the balance between antiviral defense and immunopathology during H5N1 infection. Future studies should clarify the context-dependent roles of this interaction based on the wild-type and PA-X-deficient virus, particularly how it is modulated by strain-specific virulence factors and varies across different animal models and dosing regimens. While our findings establish that PA-X binds to and enhances LncRNA#61-mediated pyroptosis, the detailed molecular consequences of this interaction remain to be fully defined. It is well known that PA-X is actively involved in host shutoff, cell death, and viral pathogenicity (79–81). However, currently, direct evidence linking its host shutoff activity to pyroptosis and viral virulence is still lacking. A critical next step is to determine whether the host shutoff activity of PA-X is required for its enhancement of LncRNA#61’s phenotype *in vivo*. To directly test this, it will be necessary to assess disease severity, pyroptosis markers, and host shutoff activity in wild-type and LncRNA#61-deficient mouse models infected with either wild-type virus or a PA-X-deficient virus, as generating a catalytically inactive PA-X mutant without disrupting the essential PA endonuclease activity required for viral replication is technically infeasible. In addition, another pivotal question emerges: is LncRNA#61 itself a substrate for PA-X-mediated cleavage? If not, does LncRNA#61 harbor intrinsic features, such as specific secondary structures or protein-binding sites that confer resistance to PA-X cleavage? Alternatively, their interaction may have a non-degradative role, such as sequestering PA-X to modulate its localization, stability, or activity against other RNA targets. Furthermore, LncRNA#61 may bind PA-X and divert it from host transcripts, attenuating global host shutoff and thereby promoting pyroptosis by fostering a pro-death environment. Crucially, elucidating whether and how this binding influences PA-X’s canonical host shutoff activity will provide profound insight into the mechanism by which a host-derived LncRNA interfaces with a viral shutoff factor to drive specific cell-fate determination.

The secretion of mature IL-1β and IL-18, which depends on inflammasome-activated GSDMD pore formation, serves to restrict viral propagation. Here, we show that LncRNA#61 enhances the expression of both cytokines *in vitro* and *in vivo*. IL-1β functions to restrict the virus through multiple mechanisms. It drives an antiviral interferon response in H1N1-infected dendritic cells (82) and contributes to viral clearance during H5N1 infection (83). It also orchestrates adaptive immunity by promoting CD4^+^ T-cell differentiation and expansion while enhancing CD8^+^ T-cell expansion and effector functions (84, 85). This is corroborated by the findings in IL-1R-deficient mice, which display impaired T-cell priming, reduced IgM production, and decreased survival during IAV infection (86). In addition, hyperactivation of the NLRP3 inflammasome further mounts a protective response by driving IL-1β-mediated neutrophil recruitment (87). As for IL-18, it functions as a key immunomodulator by orchestrating the activation of multiple immune cell types (e.g., Th1/Th2, NK, NKT, macrophages, CD8^+^ T cells, and inflammatory monocytes), thereby enhancing IFN-γ production and effector functions. By this mechanism, IL-18 exerts broad-spectrum antiviral activity against a range of pathogens across multiple infection models, such as hepatitis B virus (HBV) (88), IAV (89), HSV-2 (90), HSV-1 (91), rotavirus (RV) (92, 93), vaccinia virus (94, 95), and dengue viruses (DENV) (96). For example, IL-18 promotes antigen-specific cytokine production by CD8^+^ T cells, aiding the clearance of IAV from murine lungs (89). Beyond facilitating adaptive immunity, it directly disrupts RV replication by killing infected intestinal epithelial cells, causing the release of defective virions (92). Notably, exogenous IL-18 administration clears RV infection in mice, underscoring its therapeutic potential (93). Furthermore, IL-18-induced IFN-γ production and subsequent NOS2 induction are critical for host defense against DENV (96). Our findings suggest that LncRNA#61 enhances pyroptosis and elevates the production of IL-1β and IL-18. We hypothesize that it is pyroptosis, IL-1β, IL-18, and the membrane rupture-mediated releases of DAMPs that orchestrate an optimal anti-viral immune environment.

While the Rag-Ragulator complex is widely recognized as a nutrient sensor, emerging studies highlighted its direct regulatory role in pyroptosis (44, 55). For example, the lysosomal FLCN-FNIP2-Rag-Ragulator super-complex drives RIPK1-caspase-8-GSDMD-mediated pyroptosis during Yersinia infection (55). In parallel, a landmark study established the Ragulator-Rag-mTORC1 pathway as a key metabolic regulator of pyroptosis in macrophages, where it promotes ROS production and then enhances GSDMD oligomerization and pore formation activity (44). It is noteworthy that GSDMD pore formation requires S-palmitoylation at Cys191, a process potentiated by mitochondrial ROS (48). Our present work reveals a new paradigm in host–virus interaction by demonstrating that LncRNA#61 directs pyroptosis through activating the RagA-ROS-GSDMD axis, and by directly engaging the viral PA-X protein to amplify this antiviral response. Although the precise role of GSDMD during H5N1 influenza virus infection has remained largely unexplored, our study defines how LncRNA#61 enhances RagA-ROS-GSDMD-dependent pyroptosis to restrict viral replication both *in vitro* and *in viv*o. In future studies, we will delineate a more comprehensive regulatory network by investigating how LncRNA#61 engages the Rag-Ragulator-mTORC1-ROS axis. Specifically, based on the RagA and GSDMD-knockout mice, we will determine whether LncRNA#61-enhanced ROS oxidizes specific cysteine residues to stabilize the GSDMD pore, or alternatively, influences other GSDMD post-translational modifications.

Our transcriptomic results indicate that LncRNA#61 significantly influences lipid metabolism pathways. Unfortunately, this part of the experiment was not further explored due to time constraints. Cell death is often intertwined with cellular metabolic states, and lipid metabolism plays a role in mediating pyroptosis (42, 43). We therefore surmised that the effect of LncRNA#61 on cellular metabolism may have a link to cell death. Our future studies will also examine whether lipid metabolism, particularly through key mediators, such as ALOX5, ARA, and 15S-HETE, in turn, modulates LncRNA#61-mediated pyroptosis. These efforts will ultimately elucidate how LncRNA#61 precisely amplifies pyroptotic signaling through this integrated pathway. In addition, exploring how LncRNA#61-mediated metabolic reprogramming might influence the host’s antibacterial defense also represents a compelling and important research direction. We explicitly propose a model wherein LncRNA#61 reprograms host metabolism toward a state that is conducive to an enhanced antibacterial immune defense. For instance, we hypothesize that the observed upregulation of key enzymes (CYP4F3, CYP4F2, ALOX5, and PLA2G2A) and metabolites (15S-HETE and 9S-HODE) within the arachidonic acid metabolic pathway may enhance host defense by (i) generating microbicidal or bacteriostatic lipid mediators (such as specific HETEs and HODEs) that could directly limit bacterial survival in the niche, and/or (ii) potentiating the bactericidal activity of immune cells, like extracellular trap of neutrophils, and macrophages through elevated production of pro-inflammatory and chemotactic signals (e.g., leukotriene precursors from ALOX5 activity) that promote phagocyte recruitment and activation.

In summary, this work uncovers an antiviral strategy mediated by LncRNA#61, which potentiates pyroptosis to defend against the highly pathogenic H5N1 virus infection. We establish that LncRNA#61 suppresses viral replication by enhancing the RagA-ROS-GSDMD axis, a process synergistically augmented through interaction with the viral PA-X protein. Our findings elucidate a novel lncRNA-mediated mechanism that fine-tunes pyroptosis to combat IAV infection, providing a foundational rationale for targeting LncRNA#61 as a host-directed therapeutic strategy aimed at balancing effective immune defense against excessive tissue damage.

## MATERIALS AND METHODS

### Viruses, cells, and plasmids

To prepare viral stock, the H5N1 CK10 virus (A/Chicken/Jiangsu/k0402/2010) isolated by our lab (97), and its PA-X-deficient virus (PA-X-FS), which was generated previously (77), were first propagated in 10-day-old SPF embryonated chicken eggs at 37°C for 1–3 days. To ensure purity, the virus was then subjected to three rounds of plaque purification in CEF cells. Following purification, a seed stock was generated by amplifying the virus once in 9-day-old SPF eggs. For cell culture, Madin-Darby canine kidney cells (MDCK), human embryonic kidney cells (HEK 293T), human non-small-cell lung cancer cells (A549), and murine lung epithelial type I cells (LET-1) were grown in DMEM (Gibco, MA, USA) with 10% heat-inactivated fetal bovine serum (FBS, Gibco) at 37°C and 6% CO₂. All cell lines were used within 20 passages and verified to be mycoplasma-negative by PCR. The PA-X gene of the CK10 virus was subcloned into the expression plasmid pcDNA-3.1 as previously described (77). To enforce LncRNA#61 expression, LncRNA#61 was subcloned into pcDNA-3.1 and named LncRNA#61. Expression of LncRNA#61 was measured by qRT-PCR with the following primers: forward, 5′-CCCGTAAATCTGAAGCTAATGCA-3′; reverse, 5′-TCACTGTCACACTGGTCACTCCTA-3′.

### LNP encapsulation of LncRNA#61 and PA-X

LNPs, including LNP-LncRNA#61, LNP-PA-X (mRNA), and LNP-LncRNA#61 + PA-X, were prepared using a microfluidic mixing method. The lipid mixtures were dissolved in anhydrous ethanol at a molar ratio of 50:10:38:1.5:0.5 for ionizable lipids (SM102) (Cat. No. 06040008800, SINOPEG, Xiamen, China), 1,2-distearoyl-sn-glycero-3-phosphocholine (DSPC) (Cat. No. 06030001100, SINOPEG), and cholesterol (Cat. No. 0604001030, SINOPEG) and PEGlipid (Cat. No. 06020112402, SINOPEG) to form the organic phase (98). Then, LncRNA#61 was diluted in an acidic aqueous buffer (pH 4.0) to form the aqueous phase. The two phases were then rapidly mixed at a fixed flow-rate ratio (aqueous-to-organic =3:1) in a microfluidic chip to induce instantaneous lipid self-assembly and RNA encapsulation. The crude LNP suspension was immediately exchanged into 1 × PBS buffer (pH 7.4) via dialysis to remove ethanol and adjust to physiological conditions, and then concentrated to an RNA/DNA concentration of 0.1 mg/mL with the 30 kD Amicon Ultra Centrifugal Filters (Cat. No. UFC90309, Millipore, Massachusetts, USA). Subsequently, the LNP was sterile-filtered (0.22 μm) and characterized for size, polydispersity index (PDI), *zeta* potential, and encapsulation efficiency. After passing quality control, the formulation was aseptically aliquoted and stored at 4°C for short-term use or at −80°C for long-term preservation, with care taken to avoid repeated freeze-thaw cycles.

### Evaluation of *in vivo* antiviral effect of LNP-LncRNA#61 or LNP-LncRNA#61 + PA-X

To verify the *in vivo* delivery efficiency, mice were intramuscularly injected in the hind leg with 100 µL of LNP encapsulating 4 µg of Cy5.5-labeled LncRNA#61, PA-X, or LncRNA#61 + PA-X, respectively. The distribution of these LNPs was monitored using an IVIS Spectrum CT imaging system (PerkinElmer, Shanghai, China). Bioluminescence images were acquired from live mice at 12 h and 24 h post-inoculation, followed by *ex vivo* imaging of the isolated organs. To evaluate the antiviral effect of LNP-LncRNA#61 *in vivo*, forty-four 6-week-old female BALB/c mice were randomly assigned to four groups (*n* = 11), including MOCK, PBS, LNP, and LNP-LncRNA#61. Under anesthesia, all mice were intranasally inoculated with the CK10 virus at a dose of 15 MLD_50_ (mouse 50% lethal dose). At 6 h p.i., mice in the designated groups received a single intramuscular injection of 100 µL LNP or LNP-LncRNA#61 (each containing 4 µg of the respective component). On days 4 and 6 p.i., three mice per group were euthanized for organ collection. Tissues were processed for histopathological analysis by H&E staining, detection of viral NP expression via IFA, and viral titration in SPF eggs (determined as EID₅₀). The remaining mice (*n* = 5 per group) were monitored daily for 14 days for weight changes and survival. Mice exhibiting severe clinical signs or a weight loss ≥25% of their initial body weight were humanely euthanized and recorded as dead the following day. To minimize bias, all weight, mortality, and morbidity data were recorded by two investigators blinded to the group assignments.

To further evaluate the combined antiviral effect of LncRNA#61 and PA-X, sixty-six 6-week-old female BALB/c mice were randomly divided into six groups (*n* = 11), including MOCK, PBS, LNP, LNP-PA-X, LNP-LncRNA#61, and LNP-LncRNA#61 + PA-X. Viral challenge and the subsequent LNP treatment protocol were identical to the experiment described above, with each group receiving its corresponding LNP formulation. All subsequent procedures for sample collection, analysis, and monitoring followed the same methodology as outlined above.

### Histopathological examination

Histopathological examination was conducted as previously described (99). Briefly, lungs were harvested from euthanized mice at 4 and 6 days p.i., fixed in 4% paraformaldehyde for 48 h, and embedded in paraffin. Tissue sections (3–4 µm thick) were prepared and stained with H&E for histological analysis. Lung lesions were evaluated based on the severity of the following features: airway and alveolar cell death, inflammatory cell infiltration, hemorrhage and/or congestion, and thickening of the alveolar wall. Histopathological changes were scored on a six-point scale: 0 (normal), 1 (minimal), 2 (mild), 3 (moderate), 4–5 (marked), and 6 (severe) (Table S1).

### Immunofluorescence assay

IFA was performed on paraffin-embedded lung sections. Following deparaffinization in xylene and rehydration through a graded ethanol series, endogenous peroxidase activity was quenched with 3% H₂O₂. Antigen retrieval was performed by heating sections in 10 mM sodium citrate buffer (pH 6.0) at 95°C for 1 h. After blocking with saturated BSA for 30 min, sections were incubated with primary antibody at 37°C for 2.5 h in a humid chamber, and then visualized using an FITC-conjugated secondary antibody. Primary antibodies used were as follows: influenza NP (Cat. No. GTX125989, GeneTex, San Antonio, USA), influenza PA (Cat. No. GTX118991, GeneTex), CD163 (Cat. No. 14-1631-82, Invitrogen, CA, USA), cytokeratin (Cat. No. ab7753, Abcam, UK), cleaved GSDMD (cGSDMD, Cat. No. ab215203, Abcam), and IL-1β (Cat. No. NB120-8319, Novus Biologicals, Colorado, USA). Images were captured using a BX63 fluorescence microscope (Olympus, Tokyo, Japan) and analyzed with Image-Pro Plus 6.0 software (Media Cybernetics, Maryland, USA). For each image, the area of specific staining was measured. The measurements from all images of the same animal were averaged to obtain a single value per animal. These individual animal averages were then used as discrete data points for subsequent statistical analysis and graphical presentation.

### Immunohistochemistry

IHC was performed on paraformaldehyde-fixed tissue sections. Briefly, sections were deparaffinized in xylene and rehydrated through a graded ethanol series. Antigen retrieval was carried out by heating in citrate buffer (pH 6.0) at 121°C for 15 min. After cooling, endogenous peroxidase activity was blocked with 3% H₂O₂ for 30 min, followed by blocking with 8% non-fat milk to reduce nonspecific binding. Following three 5-min washes in TBS, sections were incubated overnight at 4°C with primary antibodies diluted in 8% skim milk against cleaved GSDMD (cGSDMD, Cat. No. ab215203, Abcam), RagA (Cat. No. 4357T, Cell Signaling Technology, MA, USA), or NLRP3 (Cat. No. 15101T, Cell Signaling Technology). Subsequently, sections were incubated with biotinylated IgG (dilution 1:250) for 1 h at room temperature. Thereafter, streptavidin-HRP was applied for 30 min. Chromogenic development was performed using 3,3'-diaminobenzidine (DAB) substrate for 5 min. Finally, the stained sections were examined and imaged under a Leica microscope, and quantitative analysis was conducted using Image-Pro Plus 6.0 software (Media Cybernetics).

### RNA extraction and purification for RNA-seq

The HEK 293T cells were seeded in 12-well plates and transfected in triplicate at 70%–80% confluence with 2 µg of either EV or the LncRNA#61 plasmid. Total RNA was extracted 24 h post-transfection using the RNeasy Mini Kit (QIAGEN, Shanghai, China). RNA integrity was verified with an Agilent 4200 Bioanalyzer, followed by on-column DNase I treatment (QIAGEN). Only RNA samples with an OD₂₆₀/₂₈₀ ratio close to 2.0 were used for the subsequent RNA-seq.

### Library preparation and sequencing for RNA-Seq

Sequencing libraries were constructed from the RNA samples using the TruSeq RNA Sample Preparation Kit (Illumina, California, USA) per the manufacturer’s instructions. Poly-A mRNA was isolated with poly-T magnetic beads and fragmented at 94°C for 8 min in the presence of divalent cations. First- and second-strand cDNA were synthesized sequentially. The cDNA fragments were then subjected to end repair, a-tailing, and adapter ligation. After purification and PCR amplification, library concentration and size distribution were assessed using a Qubit 2.0 Fluorometer and an Agilent 4200 Bioanalyzer, respectively. Finally, libraries were diluted to 10 pM, clustered on a cBot system, and sequenced on an Illumina NovaSeq platform. All procedures were performed by Shanghai Silver Crown Biomedical Technology Co., Ltd.

### Analysis of the SDE genes

Sequencing reads were preprocessed by removing adapter sequences, ribosomal RNA (rRNA) reads, short fragments, and other low-quality data. The resulting high-quality reads were aligned to the human reference genome hg38 using Tophat v2.1.0 (100), permitting up to two mismatches. Following alignment, gene expression levels were calculated in Fragments Per Kilobase of exon model per Million mapped fragments (FPKM) using Cufflinks v2.1.1 with reference annotations (101). SDE gene expression analysis was then conducted using Cuffdiff, with *P*-values adjusted for multiple testing using a false discovery rate (FDR) threshold (95). Significantly differentially expressed genes were defined by an adjusted *P*-value <0.05 and an absolute fold-change of at least 2.

### Function analysis of the SDE genes

Functional enrichment analysis of the SDE genes was performed using the DAVID database (DAVID; https://david.ncifcrf.gov) for Gene Ontology (GO) and KEGG pathways. First, expression data were log₂-transformed and median-centered using the “Adjust Data” function in *R*. Subsequently, hierarchical clustering with average linkage was applied. Enriched GO biological processes and KEGG pathways were identified with Expressing Analysis Systematic Explorer (EASE) score thresholds of *P* < 0.01 and *P* < 0.05, respectively. Higher enrichment scores and gene counts indicated greater biological relevance. Results were visualized using Java TreeView.

### Verification of SDE genes by qRT-PCR

To validate the RNA-Seq results, qRT-PCR was performed on a panel of representative differentially expressed genes involved in lipid metabolism and immune response (e.g., CYP4F3, ALOX5, NLRP3, GSDMD, IL-1β, IL-18, RagA, and MTORC1). Briefly, total RNA was isolated from transfected 293T cells with TRIzol reagent and treated with DNase I. cDNA was then synthesized from 1 μg RNA using RevertAid Premium reverse transcriptase (400 U), random primers (100 μM), and RNase inhibitor at 50°C for 30 min. Each qRT-PCR mixture was prepared with cDNA, 200 nM of each primer, and 10 μL of 2 × SYBR Green PCR Master Mix (TaKaRa, Japan). Reactions were performed in triplicate on an ABI Prism 7900 system (Applied Biosystems, USA) using the following thermal profile: 50°C for 2 min, followed by 95°C for 5 s, and 40 cycles of 95°C for 5 s and 60°C for 31 s. Melting-curve analysis was subsequently conducted to confirm amplification specificity. The relative expression of each target gene, normalized to the endogenous control β-actin, was calculated using the 2*^−^*^ΔΔCt^ method.

### Targeted metabolomic analysis of the arachidonic acid pathway using multiple reaction monitoring mass spectrometry

For targeted metabolomic analysis of the arachidonic acid pathway, multiple reaction monitoring mass spectrometry (MRM-MS) was employed. HEK 293T cells transfected with LncRNA#61 or EV (*n* = 6 per group) were homogenized in ice-cold 80% methanol containing a deuterated internal standard mixture (e.g., d8-arachidonic acid, d4-PGE₂). Following vortexing and centrifugation, the supernatant was collected, dried under nitrogen, and reconstituted in methanol-water for LC-MS/MS. Chromatographic separation was performed on a reversed-phase C18 column using a water-acetonitrile gradient (each containing 0.1% formic acid). Metabolites were ionized in negative ion mode and detected using a triple quadrupole mass spectrometer (AB SCIEX, Boston, USA) operating in MRM mode. Optimized MRM transitions, collision energies, and retention times for each target metabolite (e.g., ARA, PGE₂, LTB₄, and HETEs) were established by prior analysis of authentic chemical standards. For quantification, the peak area ratio of each analyte to its corresponding internal standard was calculated and compared against a multi-point calibration curve constructed from serially diluted standards. All data were acquired and processed using the manufacturer’s software (AB SCIEX).

### Viral growth kinetics

To assess the effect of forced expression of LncRNA#61 on viral replication, HEK 293T cells were transfected with either LncRNA#61 or an empty vector control. After 24 h, cells were infected with the influenza virus at an MOI of 0.01. Supernatants were collected at 12, 24, 36, 48, 72, and 96 h p.i. Viral titers were determined by titration on MDCK cells and calculated using the Reed-Muench method, expressed as TCID_50_/0.1 mL.

### Quantification of NP mRNA, vRNA, and cRNA

To quantify NP-specific mRNA, vRNA, and cRNA during H5N1 virus infection, LET-1 cells transfected with empty vector, LncRNA#61, PA-X, or LncRNA#61 + PA-X were infected with the H5N1 virus at an MOI of 0.1. Total RNA was extracted at 24 h post-infection using TRIzol reagent. Reverse transcription was performed with strand-specific primers: oligo (dT) for mRNA, and tagged primers complementary to the 3′ ends of vRNA (5′-AGCAAAAGCAGGGTAGATAATCACTC-3′) and cRNA (5′-AGTAGAAACAAGGGTATTTTTCTTT-3′) for detection of vRNA and cRNA, respectively. Quantitative PCR was then conducted using NP-specific primers (forward: 5′-AGAGACGGAAAATGGGTGAGAGAGC-3′; reverse: 5′-GGATCCATTCCAGTACGCACGAGAG-3′). Expression levels were normalized to β-actin and analyzed using the 2*^−^*^ΔΔCt^ method.

### Cell death analysis

To assess apoptotic and necrotic cell death, HEK 293T cells or A549 cells were transfected with LncRNA#61 or an empty vector and then infected with CK10 virus at an MOI of 2. At 12 or 24 h p.i., cells were analyzed by flow cytometry (Biolegend, San Diego, CA, USA) for annexin V single-positive (apoptotic) and annexin V/PI double-positive (necrotic/late apoptotic) populations. For PI-based death assays, LET-1 or MDCK cells in 24-well plates were transfected and then mock-infected or infected with 1 MOI of CK10 virus. During infection, propidium iodide (PI; Beyotime, Shanghai, China) was added to the medium at a concentration of 2 µg/mL. At indicated times, plates were centrifuged (400  ×  *g*, 5 min), and cell death was quantified by measuring PI fluorescence using either imaging or a plate reader (Tecan, Switzerland).

### LDH release experiment

HEK 293T, LET-1, A549 cells, or MDCK cells were plated in 24-well plates and transfected with the designated plasmids. Following transfection, cells were either maintained as uninfected controls or infected with CK10 virus at an MOI of 1. At the indicated time points post-infection, culture supernatants were harvested. LDH release into the supernatant was measured using the CytoTox 96 Non-Radioactive Cytotoxicity Assay (Promega, Madison, Wisconsin, USA) in strict accordance with the manufacturer’s guidelines.

### Determination of IL-1β and IL-18 expression

LET-1, A549, and MDCK cells were seeded in 35 mm dishes and transfected with the indicated plasmids. Following transfection, cells were either left uninfected (mock control) or infected with the H5N1 virus at an MOI of 1. Cell culture supernatants were harvested at designated time points. Concentrations of IL-1β and IL-18 were determined using commercial ELISA kits specific for the corresponding species (mouse IL-1β and IL-18 kits from Elabscience Biotechnology Co., Ltd.; canine IL-1β and IL-18 kits from Wuhan Fine Biotech Co., Ltd.) in accordance with the provided protocols. The assay was developed with TMB substrate (Sigma-Aldrich, St. Louis, MO, US), stopped with 1 M H₃PO₄ (Fisher Scientific), and absorbance was measured using a Bio-Rad 680 microplate reader. Cytokine concentrations were calculated by four-parameter logistic curve fitting performed on the online platform https://www.myassays.com/.

### Inhibition assay of the pyroptosis effector proteins

To delineate the role of specific pyroptosis effector proteins in LncRNA#61-mediated pyroptosis, LET-1 cells were transfected with either an empty vector or the LncRNA#61 expression plasmid. Cells were seeded in 48-well plates at a density of 1 × 10⁴ cells per well and cultured overnight prior to transfection. At 24 h post-transfection, cells were pretreated for 24 h with a series of concentrations of the following inhibitors: Z-VAD-FMK (pan-caspase inhibitor, Cat. No. M3143, AbMole, Shanghai, China), VX-765 (Caspase-1 inhibitor, Cat. No. HY-13205, MCE, New Jersey, USA), MCC950 (NLRP3 inhibitor, Cat. No. 210826-40-7, AbMole), Ac-DEVD-CHO (Caspase-3 inhibitor, Cat. No. HY-P1001, MCE), and disulfiram (GSDMD pore formation inhibitor, Cat. No. M3390, AbMole). After pretreatment, cells were either mock-infected or infected with the H5N1 CK10 virus at an MOI of 1. Subsequent evaluations included measurements of cell death and viral replication.

### Western blotting

Proteins were extracted from cultured cells using lysis buffer containing protease inhibitor cocktail (Cell Signaling Technology) according to the manufacturer’s protocol. Protein concentration was quantified with the bicinchoninic acid (BCA) assay (Thermo Scientific, Massachusetts, USA). Equal protein amounts were resolved by SDS-PAGE and electrophoretically transferred onto polyvinylidene fluoride (PVDF) membranes (Bio-Rad, California, USA). The membranes were blocked with 5% non-fat milk in Tris-buffered saline with 0.1% Tween-20 (TBST, pH 7.4) and subsequently probed with the primary antibodies overnight at 4°C, including anti-GSDMD (Cat. No. ab219800, Abcam), anti-RagA (Cat. No. 4357, Cell Signaling Technology), anti-Caspase-1 (Cat. No. 24232, Cell Signaling Technology), anti-NLRP3 (Cat. No. ab270449, Abcam), and anti-β-actin (Cat. No. 4970, Cell Signaling Technology). Membranes were washed three times with TBST (10 min per wash) and then incubated for 1 h at room temperature with horseradish peroxidase (HRP)-conjugated secondary antibodies (anti-mouse or anti-rabbit; Sigma). After washing, protein bands were detected using enhanced chemiluminescence (ECL) substrate (Pierce, Illinois, USA) and imaged with a FluorChem E system (ProteinSimple, Shanghai, China).

### Generation of the GSDMD- or RagA-knockout LET-1 cell line

GSDMD- or RagA-knockout LET-1 cells were generated using CRISPR-Cas9 gene editing. sgRNAs targeting GSDMD or RagA were designed with Benchling software. The following oligo sequences were used: for GSDMD, 5′-CAGCATCCTGGCATTCCGAG-3′ (sgGSDMD-1) and 5′-CAGAGGCGATCTCATTCCGG-3′ (sgGSDMD-2); for RagA, 5′-GGTTCCCCAAGAATCGGACG-3′ (sgRagA-1) and 5′-GATCAGCTGATAGACGATGC-3′ (sgRagA-2). To generate GSDMD- and RagA-knockout LET-1 cells, complementary oligonucleotides with 4-nt overhangs were annealed and ligated into BsmBI-digested LentiCRISPRv2 (Addgene, Massachusetts, USA). LET-1 cells in 24-well plates were transfected with the resulting sgRNA construct or empty vector using TurboFect reagent. After 6 h, the medium was refreshed. At 40 h post-transfection, cells were dissociated, replated into six-well plates, and selected with 3 µg/mL puromycin (medium replaced every 3 days). Following 3 weeks of selection, single clones were picked, expanded, and screened for protein loss by Western blot. At least two independent knockout clones per gene were used to assess their role in LncRNA#61-induced cell death.

### RNA immunoprecipitation

RIP assays were performed to examine the association of influenza viral proteins with LncRNA#61. The MagnaRIP RNA-Binding Protein Immunoprecipitation Kit (Cat. No. 17-700, Millipore) was used following the provided protocol. Antibodies targeting influenza PB2 (Cat. No. GTX125926, GeneTex), PB1 (Cat. No. GTX125923, GeneTex), NP (Cat. No. GTX125989, GeneTex), HA (generated in-house), and PA-X (generated in-house), as well as a control IgG (Cat. No. 12-370, Sigma), were separately conjugated to magnetic beads. The antibody-bound beads were incubated with lysates from LET-1 cells at 4°C for 24 h. Beads were then washed, and the co-precipitated RNA was recovered by proteinase K digestion and purification. The purified RNA was subjected to qPCR analysis to quantify the enrichment of LncRNA#61 and normalized to control transcripts.

### RNA pull-down assay

An RNA pull-down assay was conducted to determine whether LncRNA#61 directly interacts with the viral proteins PA-X or NP. Biotin-labeled sense (LncRNA#61) and antisense control RNAs were synthesized by *in vitro* transcription using the T7 RiboMAX Express system (Promega) with biotin-11-UTP, purified, and refolded in RNA structure buffer. Cell lysates were prepared from 293T cells expressing PA-X or NP using NP-40 lysis buffer supplemented with protease inhibitor cocktail and RNase inhibitor. For each pull-down, 2 μg of the refolded biotin-RNA was bound to streptavidin-coupled magnetic beads (Dynabeads MyOne Streptavidin C1, Thermo Scientific) for 1 h at 4°C. The RNA-coupled beads were then incubated with cell lysates overnight at 4°C with gentle rotation. Beads were washed extensively with lysis buffer, and bound proteins were eluted by boiling in SDS loading buffer. Eluates were analyzed by Western blot using anti-PA-X (generated in our lab) or anti-NP (Cat. No. GTX125989, GeneTex) antibodies. Input lysates and beads incubated without RNA were served as controls. The experiment was independently repeated three times.

### RNA fluorescence *in situ* hybridization

RNA-FISH was carried out to examine the subcellular distribution of LncRNA#61 and its potential co-localization with the viral PA-X protein. Briefly, a set of custom Stellaris FISH probes targeting LncRNA#61 and conjugated to Quasar 570 dye (Biosearch Technologies) was designed and synthesized. Cells expressing PA-X were fixed with 4% paraformaldehyde, permeabilized with 0.5% Triton X-100, and hybridized overnight at 37°C with the probes in a dedicated hybridization buffer. Post-hybridization washes were performed to remove unbound probes. For immunofluorescence detection of PA-X, cells were blocked and incubated with a primary antibody generated in-house, followed by an Alexa Fluor 488-conjugated secondary antibody. Cell nuclei were counterstained with 4′,6-diamidino-2-phenylindole (DAPI). Images were captured using a Leica DMi8 confocal microscope (Wetzlar, Germany). Control experiments utilized sense-strand probes or omitted the primary anti-PA-X antibody.

### ROS detection

Intracellular ROS was measured using the fluorescent probe 2′, 7′-dichlorofluorescein diacetate (DCFH-DA; Beyotime). Wild-type or RagA-knockout LET-1 cells were transfected with the indicated plasmids and, 24 h later, infected with CK10 virus at an MOI of 2. At 24 h post-infection, cells were incubated with 10 μM DCFH-DA for 30 min at 37°C, washed three times with PBS, and observed under a fluorescence microscope. For quantitative analysis, cells were plated in black-wall 96-well plates (Corning, New York, USA) at a density of 2  ×  10⁶ cells/mL, transfected, and infected as described. At 24 h post-infection, cells were loaded with 10 μM CM-H₂DCFDA (Thermo Scientific) in Hank’s Balanced Salt Solution (HBSS; Thermo Scientific) for 30 min. Fluorescence was immediately quantified using a Synergy H1 microplate reader (BioTek, Vermont, USA) with excitation at 493 nm and emission at 522 nm. Data are presented as indexed relative fluorescence units (iRFU), calculated as follows: (maximum RFU of test sample)/(maximum RFU of HBSS mock-control).

### Statistical analysis

Statistical analyses were performed using GraphPad Prism software (GraphPad Software, San Diego, CA). All data are presented as mean ± standard deviation (SD) from at least three independent experiments, each with a minimum sample size of *n* = 3. For comparisons between two groups, an unpaired, two-tailed Student’s t-test was used. For comparisons among three or more groups, one-way or two-way analysis of variance (ANOVA) was applied, followed by Tukey’s multiple comparison tests. Significance levels are indicated in figures as follows: *P < 0.05, **P < 0.01, ***P < 0.001, ****P < 0.0001.

## Data Availability

All primary RNA-seq data have been deposited in the Gene Expression Omnibus (GEO) database (http://www.ncbi.nlm.nih.gov/geo/info/linking.html) under the accession number GSE213790. The genomic sequences of the CK10 virus are available in GenBank under the accession numbers JQ638673 to JQ638688.

## SUPPLEMENTAL MATERIAL

Excel S1 jvi.02214-25-s0001.xlsx Targeted metabolomics analysis report of arachidonic acid by MRM.

Supplemental material jvi.02214-25-s0002.docx Fig. S1 to S5; Table S1.

ASM does not own the copyrights to Supplemental Material that may be linked to, or accessed through, an article. The authors have granted ASM a non-exclusive, world-wide license to publish the Supplemental Material files. Please contact the corresponding author directly for reuse.
