# Supplementary material for: Long noncoding RNA#61 synergizes with viral PA-X to augment pyroptosis and attenuate the virulence of highly pathogenic H5N1 influenza virus in mice
Source: J Virol. 2026 May 12;100(6):e02214-25. doi: 10.1128/jvi.02214-25 (PMC13289121; doi:10.1128/jvi.02214-25)
Supplement: Supplemental material — Fig. S1 to S5; Table S1. [file jvi.02214-25-s0002.docx]

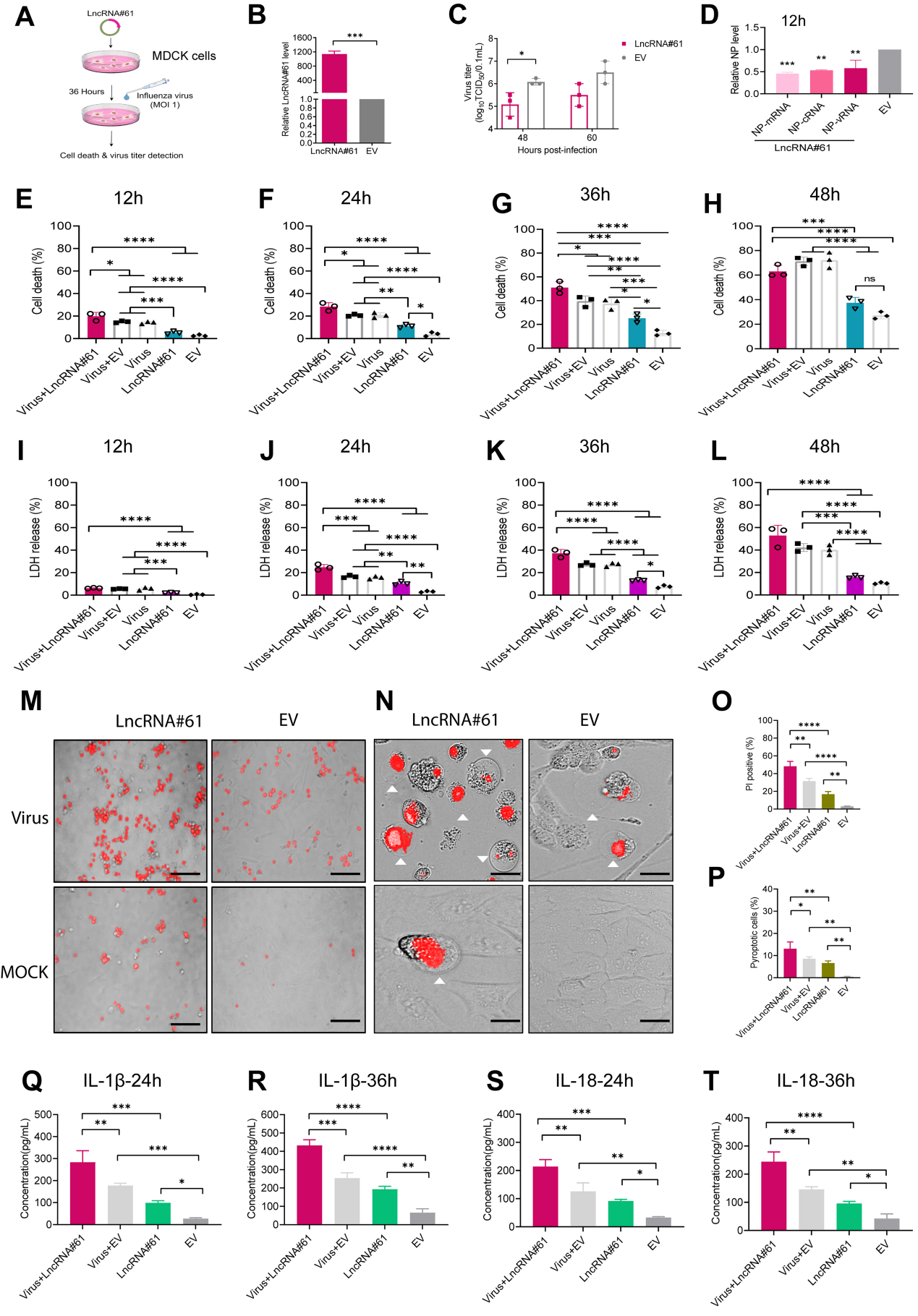
**FIG S1 LncRNA#61 promotes pyroptosis in MDCK cells.** (A) Schematic diagram of the experiment investigating the effect of the overexpression of LncRNA#61 on cell death and viral replication during H5N1 virus infection in MDCK cells. (B) Relative expression level of LncRNA#61 in MDCK cells following transfection with LncRNA#61 for 36 h. (C-D) Effect of forced LncRNA#61 expression on viral titers (C), and NP mRNA, NP cRNA, and NP vRNA levels (D) in MDCK cells. Cells were transfected with LncRNA#61 for 36 h, followed by infection with CK10 virus at an MOI of 0.01. (E-T) MDCK Cells were transfected with LncRNA#61 or EV for 36 h, followed by infection with CK10 virus at an MOI of 1. Cell death was measured at 12 h (E), 24 h (F), 36 h (G) and 48 h (H) p.i.. (I-L) LDH release was measured as shown in panels E-H at 12 h (I), 24 h (J), 36 h (K), and 48 h (L) p.i.. (M) Cell death image assessed by PI staining (red) at 36 h p.i.. Scale bar: 50µm. (N) Pyroptosis was assessed by photographing of the bubbling cells at 36 h p.i.. Red staining is represented as positive for PI dye. Scale bar: 10µm. (O) Quantitative measurement of PI fluorescence in panel M using a plate reader. (P) Quantitative measurement of pyroptosis in panel N. Quantitative measurement of pyroptosis by counting a total of 100 areas. (Q-R) IL-1β expression at 24 h (Q) or 36 h (R) p.i.. (S-T) IL-18 expression at 24 h (S) or 36 h (T) p.i.. All data are shown as means ± SD, and analyzed by unpaired and two-tailed Student’s *t*-test (Fig. S1B), two-way (Fig. S1C) or one way ANOVA with Tukey’s multiple comparison test (Fig. S1D through T),*p < 0.05, **p < 0.01, ***p < 0.001, ****p < 0.0001.


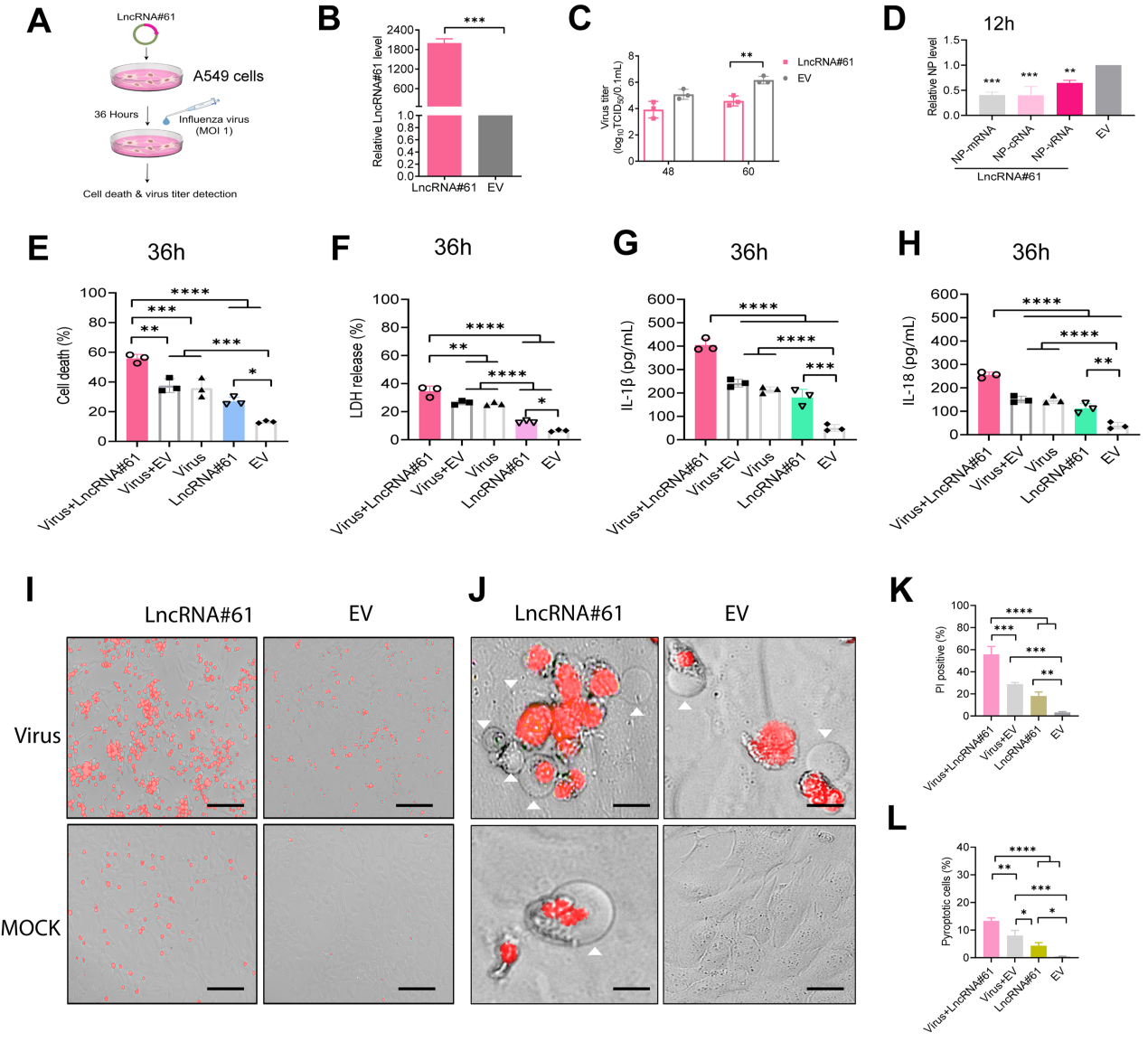
**FIG S2 LncRNA#61 promotes pyroptosis in human A549 cells.** (A) Schematic diagram of the experiment investigating the effect of the overexpression of LncRNA#61 on cell death and viral replication during H5N1 virus infection in A549 cells. (B) Relative expression level of LncRNA#61 in A549 cells following transfection with LncRNA#61 for 36 h. (C-D) Effect of forced LncRNA#61 expression on viral titers (C), and NP mRNA, NP cRNA, and NP vRNA levels (D) in A549 cells. Cells were transfected with LncRNA#61 for 36 h, followed by infection with CK10 virus at an MOI of 0.01. (E-H) A549 Cells were transfected with LncRNA#61 or EV for 36 h, followed by infection with CK10 virus at an MOI of 1. (E) Cell death was measured at 36 h p.i.. (F) LDH release was measured at 36 h p.i.. (G) IL-1β expression at 36 h p.i. (H) IL-18 expression at 36 h p.i. (I) Cell death image assessed by PI staining (red) at 36 h p.i.. Scale bar: 50µm. (J) Pyroptosis was assessed by photographing of the bubbling cells (indicated by white triangle) at 36 h p.i.. Red staining is represented as positive for PI dye. Scale bar: 10µm. (K) Quantitative measurement of PI fluorescence in panel I using a plate reader. (L) Quantitative measurement of pyroptosis in panel J. Quantitative measurement of pyroptosis by counting a total of 100 areas. All data are shown as means ± SD, and analyzed by unpaired and two-tailed Student’s *t*-test (Fig. S2B), two-way (Fig. S2C) or one way ANOVA with Tukey’s multiple comparison test (Fig. S2D through L),*p < 0.05, **p < 0.01, ***p < 0.001, ****p < 0.0001.


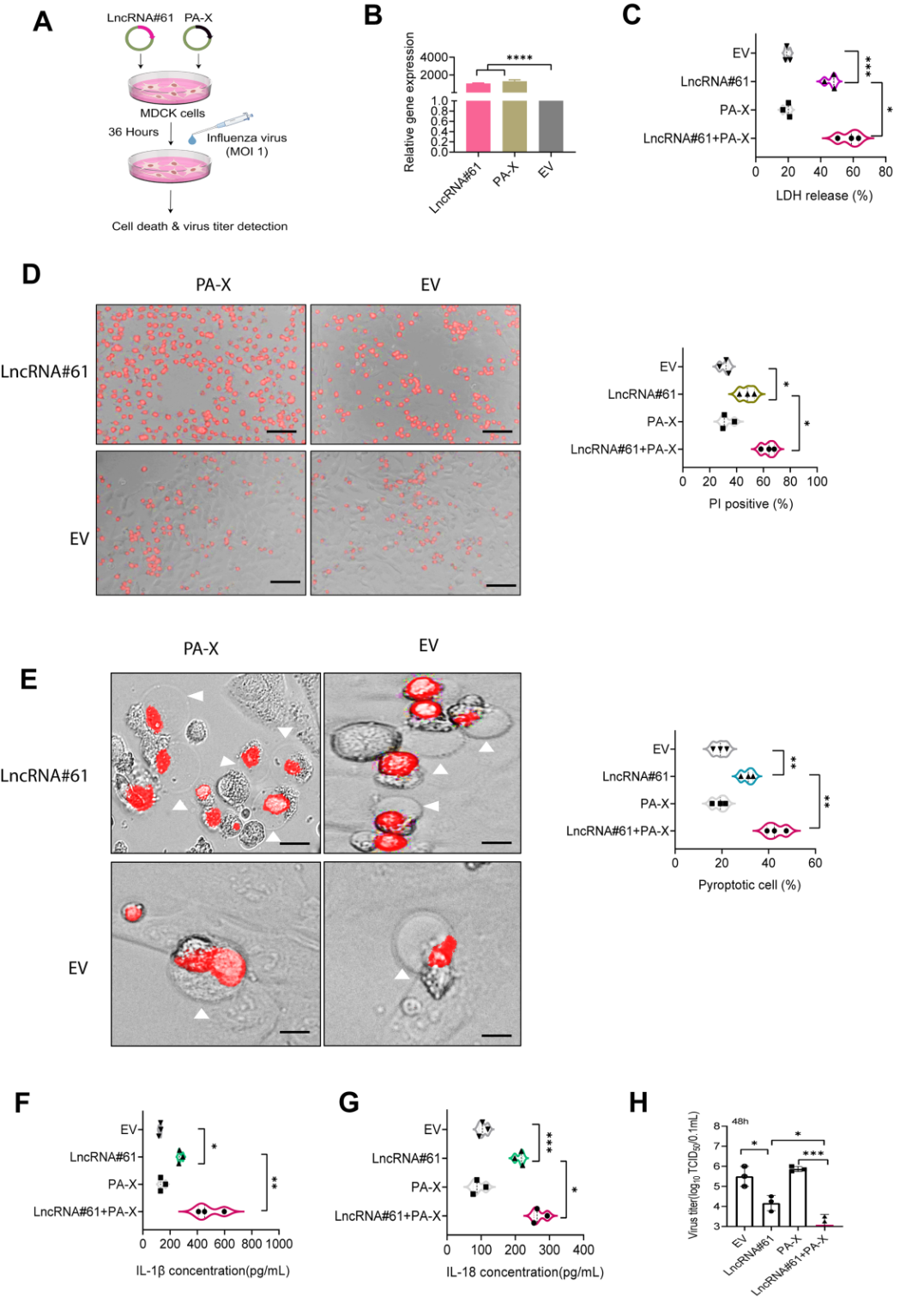


**FIG S3 LncRNA#61-PA-X interaction promotes pyroptosis in MDCK cells.** (A) Schematic of the procedure for detecting cell death and viral titer in MDCK cells through forced expression of LncRNA#61/PA‑X and subsequent viral infection. (B) Relative expression of LncRNA#61 and PA-X were determined by qRT-qPCR. (C) LDH release of the cells at 36 h p.i.. (D) PI staining (red) images at 36 h p.i.. Scale bar: 50 µm. Quantitative measurement of PI fluorescence using a plate reader. (E) Pyroptosis was assessed by photographing of the bubbling cells at 36 h p.i.. Red staining is represented as positive for PI dye. Scale bar: 10 µm. Quantitative measurement of pyroptosis by counting a total of 100 areas. (F) IL-1β expression at 36 h p.i.. (G) IL-18 expression at 36 h p.i.. (H) Effect of over expression of LncRNA#61 and PA-X on viral replication at 48 h p.i.. All data are shown as means ± SD, and analyzed by unpaired and two-tailed Student’s *t*-test (Fig. S3B), or one way ANOVA with Tukey’s multiple comparison test (Fig. S3C through H),*p < 0.05, **p < 0.01, ***p < 0.001.


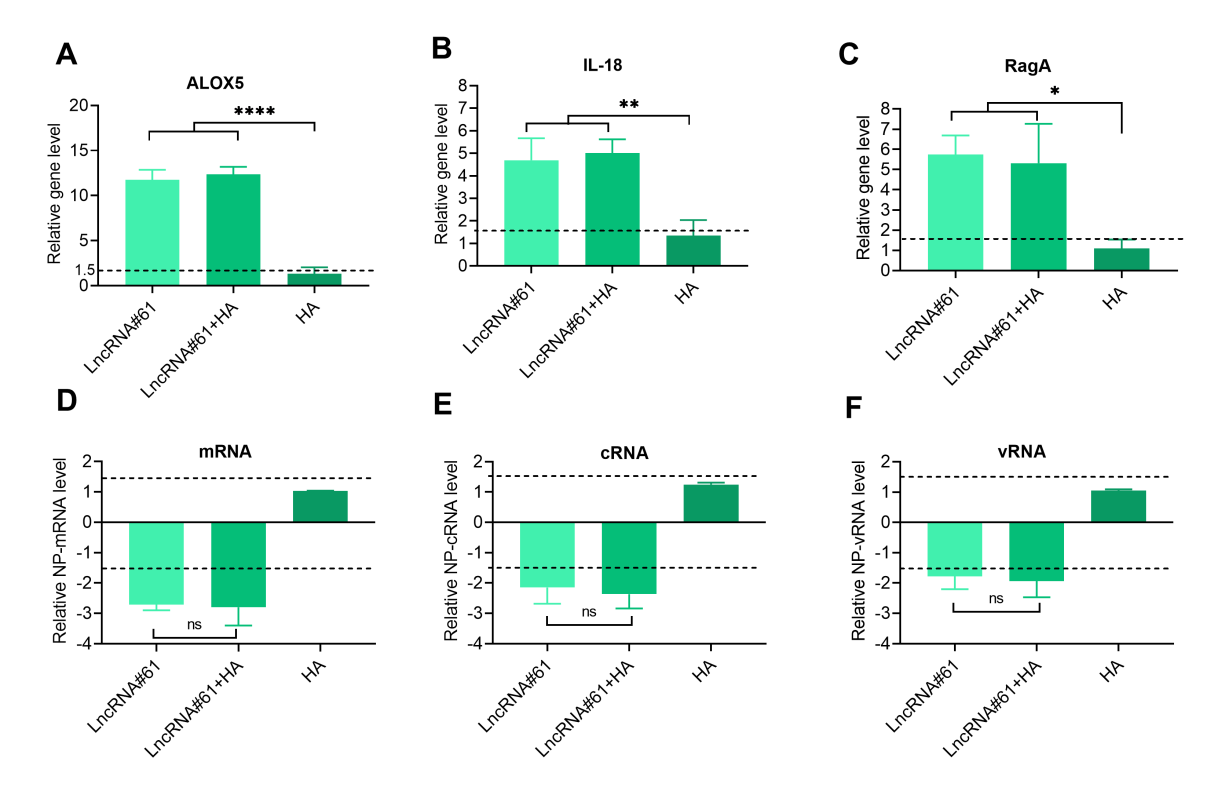
**FIG S4 Viral HA protein has no effect on the antiviral activity of LncRNA#61 in LET-1 cells.** (A-C) Effect of forced expression of LncRNA#61, HA, or HA combined with LncRNA#61 on the levels of ALOX5 (A), IL-18 (B), and RagA (C). (D-F) Effect of forced expression of LncRNA#61, HA, or HA combined with LncRNA#61 on the levels of viral NP mRNA (D), NP cRNA (E) and NP vRNA (F) at 12 h p.i.. All data are shown as means ± SD, and analyzed by one way ANOVA with Tukey’s multiple comparison test,*p < 0.05, **p < 0.01, ****p < 0.0001.


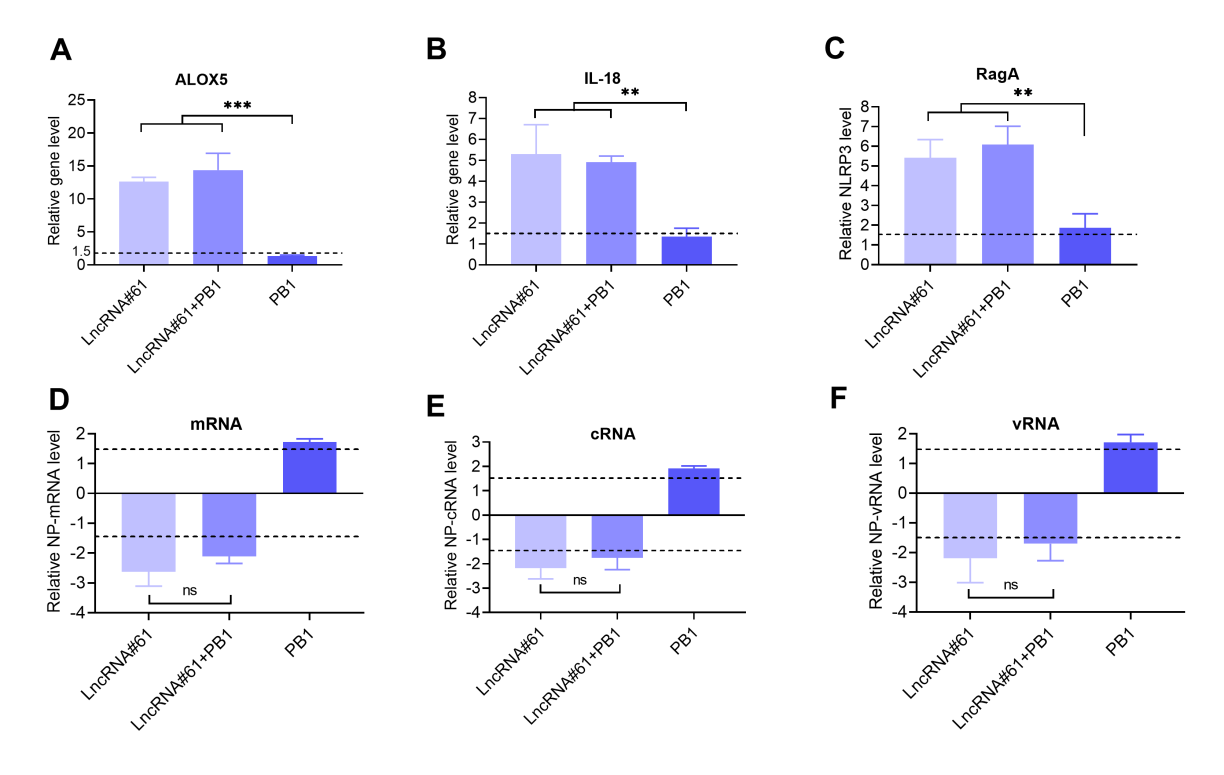
**FIG S5 Viral PB1 protein has no effect on the antiviral activity of LncRNA#61 in LET-1 cells.** (A-C) Effect of forced expression of LncRNA#61, or PB1, or PB1 combined with LncRNA#61 on the expression of ALOX5 (A), IL-18 (B), and RagA (C). (D-F) Effect of forced expression of LncRNA#61, PB1, or PB1 combined with LncRNA#61 on the levels of viral NP mRNA (D), NP cRNA (E) and NP vRNA (F) at 12 h p.i.. All data are shown as means ± SD, and analyzed by one way ANOVA with Tukey’s multiple comparison test, **p < 0.01, ***p < 0.001.

| Histologicalscore | Airway and alveolar cell necrosis | Cell infiltration | **Hemorrhage or congestion** | **Broadening of the pulmonary alveoli interval** |
| --- | --- | --- | --- | --- |
| Score 0 | Normal lung | Normal lung | Normal lung | Normal lung |
| Score 1 | Airway epithelial cells necrosis limited in one lobe | Infiltration cells only seen in peribronchiolar area or alveolar wall in one lobe | Mild and limited in one lobe | Mild broadening and limited in one lobe |
| Score 2 | Airway epithelial cell necrosis in more than one lung lobes | A few immune cells (1-5 cells) in alveolar space which located in focal area of one lobe | Moderate and limited in one lobe | Moderate broadening and limited in one lobe |
| Score 3 | Airway epithelial cell necrosis in more than one lung lobes, with luminal cell debris | More immune cells (10-20 cells) in alveolar space which located in focal area of one lobe | Severe and limited in one lobe | Severe broadening and limited in one lobe |
| Score 4 | Airway epithelial cell necrosis in more than one lung lobes; and with small area of alveolar wall collapse | A lot of immune cells infiltration in alveolar space which can be seen in more than one lobe | Mild and in more than one lung lobes | Mild broadening and in more than one lung lobes |
| Score 5 | Airway epithelial cell necrosis in more than one lung lobes; and with large area of alveolar wall collapse | Moderate diffuse immune cell infiltration in alveolar space | Moderate and in more than one lung lobes | Moderate broadening and in more than one lung lobes |
| Score 6 | Airway epithelial cell necrosis in more than one lung lobes, and alveolar wall collapse in more than one lobes | Severe diffuse immune cell infiltration in  alveolar space | Severe and in more than one lung lobes | Severe broadening and in more than one lung lobes |

Table S1. Histopathological scoring criteria
